# Beyond Planar: Enhanced Performance of Hollow Fiber Dielectric Elastomer Actuators

**DOI:** 10.1002/advs.202504803

**Published:** 2025-06-27

**Authors:** Sina Jafarzadeh, Anne Ladegaard Skov

**Affiliations:** ^1^ Danish Polymer Center Department of Chemical and Biochemical Engineering Technical University of Denmark Kgs. Lyngby 2800 Denmark

**Keywords:** dielectric elastomer actuators (DEAs), electro‐mechanical modeling, finite element modeling (FEM), hollow fiber actuators, hollow fiber dielectric elastomer actuators (HFDEAs)

## Abstract

Hollow fiber dielectric elastomer actuators (HFDEAs) offer several advantages over their conventional counterpart, planar dielectric elastomer actuators (DEAs). Due to their simple shape, flexibility, and conformability, HFDEAs are promising candidates for complex applications within soft robotics. This paper offers a comprehensive comparison between the actuation behavior of planar and HFDEAs using both analytical and numerical models. An electro‐mechanical model establishes analytical correlations between the applied voltage and resulting strain. The results from the simplified model are subsequently compared with a numerical model in COMSOL Multiphysics, where simulations are run in more realistic conditions. Supporting experiments are conducted on HFDEAs with different geometries to validate the model. A geometric factor, β, is introduced to account for the influence of geometric parameters on actuator performance. The results show that HFDEAs exhibit higher strain compared to planar films. Among the different fiber geometries, those with smaller internal diameters and thinner walls exhibit higher axial strain and holding force while using the least amount of material. This study highlights the advantages of hollow fiber DEAs compared to their planar counterparts, especially in applications where lighter, more efficient structures with greater strain capabilities are essential.

## Graphical abstract

This study presents a comprehensive analysis of hollow fiber dielectric elastomer actuators, highlighting their superior strain performance compared to planar actuators. By combining analytical modeling, numerical simulations, and experiments, it identifies optimal fiber geometries that maximize actuation efficiency. The findings support dielectric hollow fibers as lightweight, high‐strain alternatives for soft robotics and advanced actuation systems.

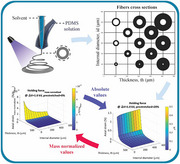

## 1 Introduction

Dielectric elastomer actuators (DEAs) hold great promise due to their lightweight nature, high flexibility, and silent operation with no need for gearing. They consist of an elastomer membrane placed between two flexible electrodes.^[^ ^1^ ^]^ The elastomers undergo mechanical deformation when a voltage is applied over the electrodes. DEAs present exceptional characteristics, such as initial actuation stresses over 1000%,^[^ ^2^ ^]^ energy densities exceeding 3 J/g,^[^ ^3^ ^]^ response times below 1 ms,^[^ ^4^ ^]^ and theoretical efficiencies reaching up to 90%.^[^ ^5^ ^]^ Their inherent softness, flexibility, and improved conformability have made them ideal candidates for artificial muscles and soft robotics.^[^ ^6^, ^7^, ^8^ ^]^

**Figure 1:**
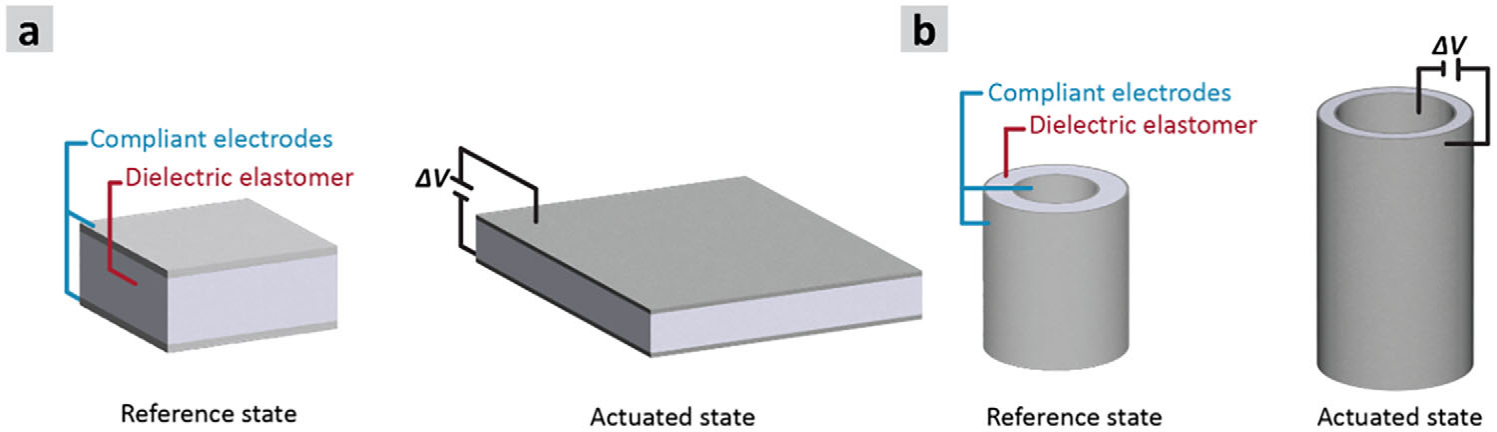
Schematic comparison between planar film and hollow fiber dielectric elastomer actuators (HFDEAs). a) The planar film actuator is shown in its rest state (left) and actuated state (right), demonstrating compression along thickness and expansion in the planar dimensions. b) The HFDEA is shown in its rest state (left) and actuated state (right), highlighting axial elongation and radial expansion due to the higher electrostatic pressure of the inner electrode compared to the outer.

DEAs can be fabricated in different configurations, most commonly relying on the planar configuration that is then stacked or folded.^[^ ^9^ ^]^ The fiber format DEA offers new possibilities due to its ease of integration.^[^ ^10^, ^11^ ^]^ **Figure** **1** demonstrates the schematic for the two configurations of DEAs, including details on the changes in length, cross‐section, and thickness before and after applying a voltage. Several preparation techniques have been studied over the years to enhance the fabrication throughput of the DEAs and to improve their functionality. For instance, roll‐to‐roll manufacturing^[^ ^12^ ^]^ and automated sequential stacking procedures^[^ ^13^ ^]^ have been successfully used to fabricate planar DEAs on small and large scales; however, these methods fall short in achieving intricate 3D structures and postures.^[^ ^8^ ^]^ On the other hand, recent advancements in fiber‐shaped DEAs—inspired by biological muscles^[^ ^8^, ^14^, ^15^, ^16^, ^17^ ^]^ with potential scaleup capabilities—have significantly contributed to the progress of more complex systems, such as active textiles,^[^ ^18^, ^19^ ^]^ exoskeletons, artificial arms^[^ ^20^, ^21^, ^22^, ^23^ ^]^ and grasping systems,^[^ ^24^, ^25^ ^]^ applications for which flexibility and conformability are of great importance. Recent studies have advanced DEA research through computational approaches. Data‐driven methods now enable rapid prediction of actuator performance and energy efficiency using machine learning and minimal experimental data.^[^ ^26^, ^27^ ^]^

Existing theoretical models have only focused on examining the actuation behavior of DEAs in the planar configuration, mainly from a thermodynamic point of view, or focusing on instabilities.^[^ ^28^, ^29^, ^30^, ^31^, ^32^, ^33^, ^34^, ^35^ ^]^ No comprehensive exploration and comparison between the planar and hollow fiber configurations have been reported. In this study, we first adopt an analytical approach to compare the strain actuation performance in planar films versus hollow fiber DEAs (HFDEAs) to understand better how different these two configurations perform regarding strain efficiency. We use electromechanical modeling to establish the analytical relationships between the applied voltage and the resulting strains. This analytical analysis entails several simplifying assumptions, such as no pre‐stretch used, incompressibility of the elastomer, and actuation within the linear regime, as predicted by Hookean behavior, with Young's modulus (*Y*) as the sole parameter coupling the strain to the stress. Subsequently, we developed a numerical model using COMSOL Multiphysics to investigate the performance of HFDEAs under more realistic conditions than the analytical model provides. The numerical model is based on the experimental values for our material properties, and the predicted actuation results from the model are further validated through actuation experiments. This predictive tool enables the investigation of parameters that are easy to measure and analyze and those that are difficult to measure experimentally for these small‐sized fibers. For example, the axial actuation strain is practical to test experimentally in the lab; thus, these measurements were conducted to validate our model. However, radial strains and holding force are challenging to measure. The holding force is the greatest force the actuator can apply to the surroundings when its deformation is restricted. These features have been shown to play critical roles in determining the overall performance of DEAs in soft robotics.^[^ ^36^, ^37^, ^38^ ^]^ Our model is shown to be capable of predicting optimal geometric dimensions to improve HFDEAs’ actuation strain and holding force, hence serving as a tool to enhance the design and performance of actuators.

## 2 Methodology

### 2.1 Analytical Principles of Actuation Modeling

When a voltage is applied over a DEA, two phenomena happen as a result of charges on the electrodes: a) an attractive force between the two layers due to the opposite charges on each electrode resulting in the compression of the sandwiched elastomer, and b) an electrostatic repulsion on the electrodes and the attached elastomer due to the similar charges on each electrode. Both of these forces contribute to the deformation of the elastomer when voltage is applied. These physical phenomena can be described mathematically to establish a relationship between the deformation of the elastomer, expressed as strain, and the voltage applied to the elastomer.

To create analytical relationships between the applied voltage and the resulting actuation strains in our DEAs, we employed electromechanical modeling using the stored electrostatic energy (*U*) in the system. The concept of leveraging changes in stored electrostatic energy in a capacitor was first introduced by Pelrine and Kornbluh in 1998 for planar DEAs^[^ ^32^ ^]^ and has been adapted for use in cylindrical configurations.^[^ ^33^, ^39^ ^]^ The common methodology of the given references is based on the electrically induced pressure in the thickness direction by deriving the total differential of electrostatic energy with respect to the electrode area and the film thickness.

Following this concept, the electromechanical analysis is conducted through a sequential two‐step process rather than simultaneously solving the equations to avoid complications from multiple interdependent variables. The first step involves calculating the stored electrostatic energy, from which we derive the electrostatic forces and stresses the electrodes exert. The electrostatic attraction pushes the electrodes toward each other while the stiffness of the elastomer resists this deformation. In the second step, the stress generated by the electrostatic force is treated as the mechanical stress acting on the elastomer, causing it to strain. Also, we assume constant electrostatic pressure from the electrodes during the actuation. This simplification enables the calculation of electrostatic pressures solely based on the initial geometric dimensions of the actuator.

Additionally, we assume that the elastomer is not prestretched, which, however, is a methodology commonly used to enhance actuation performance. With this assumption, we consider all elastic strain in the elastomer to be generated solely by the applied electrostatic energy, simplifying the analysis further. For small strains, the elastic properties of the elastomer are assumed to be linear and purely elastic, meaning the stress can be described by Hooke's law which predicts the stress to be linearly dependent on the applied strain.

**Figure 2:**
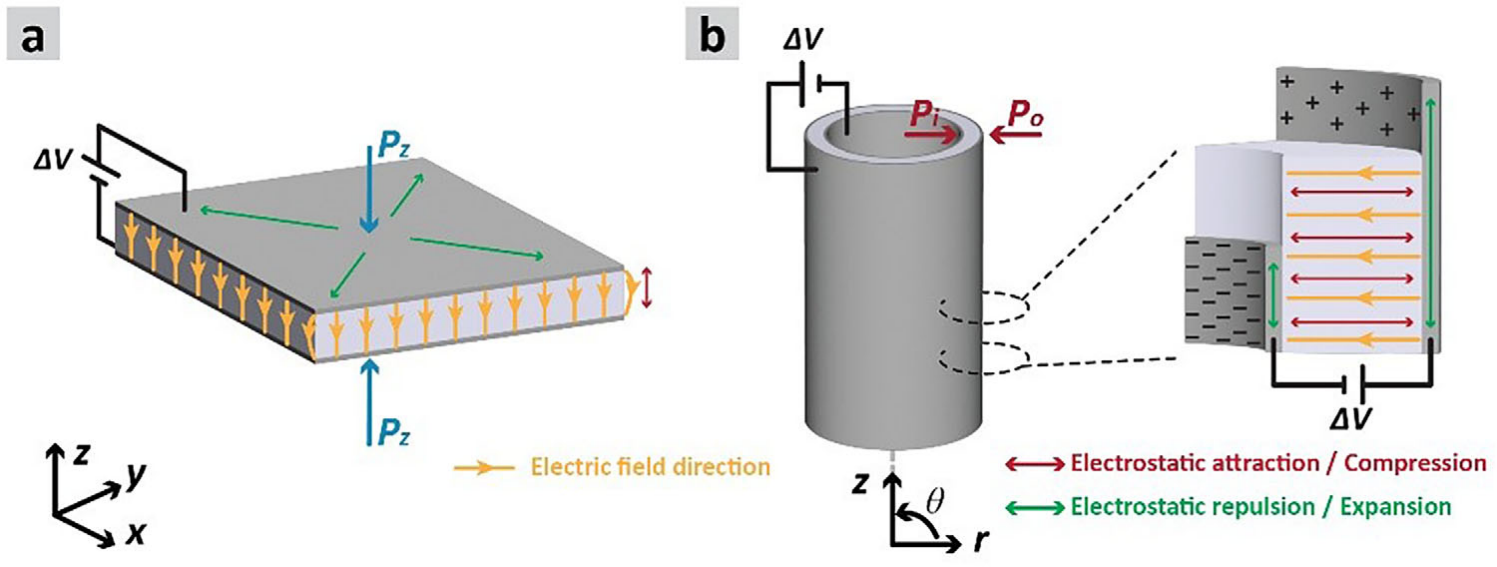
Working principle of dielectric elastomer actuators: The electrostatic pressures applied on the electrodes a) in the planar and b) in the hollow fiber format.

The electrodes are assumed to be highly conductive, very thin, and fully compliant with no contributions to the elastic properties of the DEA; therefore, their mechanical and electrical resistances are disregarded.^[^ ^40^ ^]^ It is also assumed that the applied charges are evenly distributed across the electrodes and that the resulting electric field between them has components that are normal only to the electrode area. Edge effects at the boundaries of the electrodes are thus neglected. In addition, the electrodes are assumed to be perfectly connected to the dielectric material. As described earlier, the DEA is assumed to be incompressible, isotropic, and exhibiting linear elasticity. These assumptions simplify the complex relations and help focus on the primary behavior of the system through a more straightforward calculation. To simplify the notations, we adopt ε to abbreviate the mathematical expression ε  = ε_0_ ε_*r*_, where ε_0_ is the vacuum permittivity and ε_*r*_ is the relative dielectric permittivity of the elastomer. **Figure** **2** illustrates the working principles of DEAs and electrostatic pressure distribution on the electrodes in both planar and hollow fiber formats. In Figure 2a, the planar configuration is shown where the x‐ and y‐axis represent the in‐plane coordinates, and the z‐axis represents the out‐of‐plane coordinates along the film thickness. In Figure 2b, the geometry of the hollow fiber format is presented, where the 𝜃‐axis represents the circumferential coordinate, the 𝑟‐axis represents the radial coordinate, and the *l*‐axis represents the axial coordinate along the length of the hollow cylinder.

In the following sections, the governing equations for both planar and HFDEAs are presented.

#### 2.1.1 Planar Dielectric Actuators

To describe the electromechanical behavior of DEAs, Pelrine et al.^[^ ^32^ ^]^ used the change in the stored electrostatic energy (*U*) in a capacitor for planar DEAs. The governing equations for the planar configuration are briefly summarized in Equations (1)–(5) to establish the methodology in a well‐known system. For additional details, refer to Section S1 (Supporting Information).

Generally, the stored electrostatic energy (*U*) can be expressed by the capacitance (*C*) and the applied voltage (Δ*V*), according to:

(1) $$U=\frac{C\Delta V^{2}}{2}\;$$

The stored electrostatic energy (*U*) represents the work performed on the DEA by the electric field. As a result, the DEA increases the electrostatic force (*F*) exerted on the electrodes. This force can be determined by taking the first derivative of the stored electrostatic energy with respect to the displacement in the direction of the electric field along the elastomer's thickness (*z*). For planar actuators, the electrostatic force can be expressed as:

(2) $$F_{z}=-\frac{\partial U}{\partial z}$$

In response to the applied voltage (Δ*V*), the electrostatic pressures (*P*) on each electrode can be determined by the electrostatic force acting between the electrodes divided by the respective area of the electrode (A):

(3) $$P_{z}=-\frac{1}{A}\frac{\partial U}{\partial z}$$

In equilibrium, with no pre‐stretching, and under small strain conditions, the electrostatic pressure from the electrodes equals the stress exerted by the elastomer (elastic pressure). To simplify the equations (as expressed in detail in Section S1, Supporting Information), the actual dimension of the film (z) can be approximated by the initial thickness (𝑡ℎ). This small strain assumption avoids complications arising from higher‐order nonlinearities in the material response but first of all simplifies the mathematical treatment to a great extent. Considering the thickness (th) and Young's modulus (Y) of the dielectric elastomer, the strain (*s*) in each direction can be expressed as:

(4) $$s_{z}=-\frac{P_{z}}{Y}=-\frac{\varepsilon }{Y}{\left(\frac{\Delta V}{th}\right)}^{2}$$

(5) $$s_{x}=s_{y}=\frac{1}{2}\;\frac{\varepsilon }{Y}{\left(\frac{\Delta V}{th}\right)}^{2}$$

In other words, the thickness strain (s_z_) scales quadratically with the inverse thickness, which becomes the sole, tunable geometrical parameter for the planar dielectric elastomer actuator.

#### 2.1.2 Hollow Fiber Dielectric Actuators

A HFDEA can be modeled as a hollow cylindrical capacitor. The radial coordinate, 𝑟, spans from the internal radius (*r_i_*) to the external radius (*r*_e_), defining the thickness of the hollow cylinder as *r*_e_ − *r_i_*. The electric field is oriented in the radial direction, corresponding to the 𝑟‐axis. The cylindrical geometry leads to a nonlinear distribution of the electric field in the radial direction and imposes additional mathematical complications compared to the planar configuration. Although in practice the electrostatic pressure varies as the fiber deforms, as mentioned earlier in this section, it is assumed that a constant electrostatic pressure from the electrode is exerted during the actuation. This assumption allows us to bypass the intermediate steps of the involved pressure increase and deformation, simplifying the calculation of these pressures by approximating the thickness, based on the initial geometric dimensions of the actuator. Additionally, to reduce the number of variables and enable a direct analytical expression for actuation strain, we assume that the fibers radially expand perfectly at the two fiber ends, similar to the middle of the fiber, thereby ignoring any edge effects from the electric field applied.

Similar to hollow cylinder capacitors, the capacitance of HFDEAs can be defined using the same principles. The capacitance is the ratio of the charge (*Q*) on each electrode divided by the potential difference (Δ*V*) between the two cylindrical electrodes. This relationship can be expressed as:

(6) $$C=\frac{Q}{\Delta V}\;$$

Using Gauss's law, for a cylindrical shell of radius (𝑟), length (𝑙), and charge (𝑄), the electric field (𝐸) at any point inside the dielectric elastomer is given by:

(7) $$E=\frac{Q}{2\pi \varepsilon lr}\;$$

The voltage difference between the elastomer boundaries, defined by the internal radius (*r_i_*) and the external radius (*r_e_*), is calculated as the integral of the electric field over the distance between these two points. The boundaries 𝑟_𝑖_ and 𝑟_𝑒_ correspond to the limits of integration, representing the cylindrical shell's starting point (internal radius) and the endpoint (external radius). Therefore, the voltage difference is expressed as:

(8) $$\Delta V=\int_{r_{i}}^{r_{o}}E\;dr=\int_{r_{i}}^{r_{e}}\frac{Q}{2\pi \varepsilon lr}dr=\frac{Q}{2\pi \varepsilon l}\;ln\left(\frac{r_{e}}{r_{i}}\right)$$

By rearranging Equation 8 to solve for 𝑄 and substituting it into the definition of capacitance (Equation 6), we get:

(9) $$C=\frac{2\pi \varepsilon l}{ln\left(\frac{r_{e}}{r_{i}}\right)}\;$$

The expression for the stored electrostatic energy (*U*) in the fiber format can be expressed by inserting Equation 9 into Equation 1 which gives the following expression:

(10) $$U=\frac{\pi \varepsilon l\Delta V^{2}}{ln\left(\frac{r_{e}}{r_{i}}\right)}\;$$

In an electric field, the electrostatic force can be described as the negative gradient of stored electrostatic energy (potential energy). The negative sign indicates that the force acts toward decreasing potential energy. Thus, the electrostatic force (*F*) can be expressed as:

(11) $$\vec{F}=-\nabla U$$

Here *U* is a function of three geometrical variables, 𝑟_𝑖,_ 𝑟_𝑒,_ and *z*. Therefore, the differential change in stored energy *dU* can be calculated as:

(12) $$dU=\frac{\partial U}{\partial r_{i}}\;dr_{i}+\frac{\partial U}{\partial r_{e}}dr_{e}+\frac{\partial U}{\partial l}dl$$

The magnitude of the gradient (|∇*U*|) can be determined as follows:

(13) $$\left|\nabla U\right|=\sqrt{{\left(\frac{\partial U}{\partial r_{i}}\right)}^{2}+{\left(\frac{\partial U}{\partial r_{e}}\right)}^{2}+{\left(\frac{\partial U}{\partial l}\right)}^{2}}\;$$

By using Equation 10, we can express the partial derivatives as:

(14) $$\frac{\partial U}{\partial r_{i}}=\frac{\pi \varepsilon l\Delta V^{2}}{r_{i}{\left(ln\left(\frac{r_{e}}{r_{i}}\right)\right)}^{2}}\;$$

(15) $$\frac{\partial U}{\partial r_{e}}=-\frac{\pi \varepsilon l\Delta V^{2}}{r_{e}{\left(ln\left(\frac{r_{e}}{r_{i}}\right)\right)}^{2}}$$

(16) $$\frac{\partial U}{\partial l}=\frac{\pi \varepsilon \Delta V^{2}}{ln\left(\frac{r_{e}}{r_{i}}\right)}\;$$

The assumption of incompressibility of the elastomer requires that its volume (*Vol*) remains constant during deformation:

(17) $$d\left(Vol\right)=0$$

For the hollow cylinder geometry, the volume is determined from the cross‐section area and the length, and, thus, the change of the volume can be written as:

(18) $$d\;\left(\pi \left({r_{e}}^{2}-{r_{i}}^{2}\right)l\right)=0$$

By doing a partial integration, we get:

(19) $$\left({r_{e}}^{2}-{r_{i}}^{2}\right)dl+\left(2r_{e}dr_{e}-2r_{i}dr_{i}\right)l=0$$

Finally, solving for *dl*, we find the change in length as:

(20) $$dl=\frac{-2\left(r_{e}dr_{e}-r_{i}dr_{i}\right)l}{\left({r_{e}}^{2}-{r_{i}}^{2}\right)}\;$$

This equation shows how any expansion or contraction in the radial directions 𝑟_𝑒_ and 𝑟_𝑖_ must be compensated by a corresponding change in the length (𝑙) to maintain the overall volume of the cylinder. This incompressibility condition provides a relationship among the three variables 𝑟_𝑖,_ 𝑟_𝑒_, and 𝑙, allowing us to express the change in stored energy *dU* as a function of only two of these variables.

By combining the expressions derived in Equation 12 through Equation 16 and Equation 20, the stored electrostatic energy and the resulting electrostatic force can be expressed solely by the radii of the internal and external electrodes:

(21) $$\begin{array}{cc} dU & =\frac{\pi \varepsilon l\Delta V^{2}}{r_{i}{\left(ln\left(\frac{r_{e}}{r_{i}}\right)\right)}^{2}}\;dr_{i}+-\frac{\pi \varepsilon l\Delta V^{2}}{r_{e}{\left(ln\left(\frac{r_{e}}{r_{i}}\right)\right)}^{2}}dr_{e}\\ & \;+\frac{\pi \varepsilon \Delta V^{2}}{ln\left(\frac{r_{e}}{r_{i}}\right)}\frac{-2\left(r_{e}dr_{e}-r_{i}dr_{i}\right)l}{\left({r_{e}}^{2}-{r_{i}}^{2}\right)} \end{array}$$

After some simplifications, finally, we arrive at the following form:

(22) $$\begin{array}{ccc} dU & = & \frac{\pi \varepsilon l\Delta V^{2}}{{\left(\ln\left(\frac{r_{e}}{r_{i}}\right)\right)}^{2}r_{i}r_{e}\left({r_{e}}^{2}-{r_{i}}^{2}\right)}\times \left(\left({r_{e}}^{2}-{r_{i}}^{2}+2{r_{i}}^{2}\ln\left(\frac{r_{e}}{r_{i}}\right)\right)r_{e}dr_{i}\right.\\ & & \left.-\left({r_{e}}^{2}-{r_{i}}^{2}+2{r_{e}}^{2}\ln\left(\frac{r_{e}}{r_{i}}\right)\right)r_{i}dr_{e}\right) \end{array}$$

The magnitude of the gradient (|∇*U*|) can be determined from $\sqrt{{\left(\nabla U\right)}^{2}}$. (∇*U*)^2^ can be calculated by summing the squares of the partial derivatives with respect to the internal and external radii:

(23) $$\begin{array}{ccc} \left|\nabla U\right| & = & \frac{\pi \varepsilon l\Delta V^{2}}{{\left(\ln\left(\frac{r_{e}}{r_{i}}\right)\right)}^{2}r_{i}r_{e}\left({r_{e}}^{2}-{r_{i}}^{2}\right)}\\ & & \times \;\sqrt{{r_{e}}^{2}{\left({r_{e}}^{2}-{r_{i}}^{2}+2{r_{i}}^{2}\ln\left(\frac{r_{e}}{r_{i}}\right)\right)}^{2}+{r_{i}}^{2}{\left({r_{e}}^{2}-{r_{i}}^{2}+2{r_{e}}^{2}\ln\left(\frac{r_{e}}{r_{i}}\right)\right)}^{2}} \end{array}$$

By expanding the expression, we obtain:

(24) $$\begin{array}{ccc} \left|\nabla U\right| & = & \frac{\pi \varepsilon l\Delta V^{2}}{{\left(\ln\left(\frac{r_{e}}{r_{i}}\right)\right)}^{2}r_{i}r_{e}\left({r_{e}}^{2}-{r_{i}}^{2}\right)}\\ & & \times \;\sqrt{\begin{array}{c} {r_{i}}^{6}+{r_{e}}^{6}-{r_{i}}^{2}{r_{e}}^{4}-{r_{e}}^{2}{r_{i}}^{4}+8\ln\left(\frac{r_{e}}{r_{i}}\right)\left({r_{e}}^{2}-{r_{i}}^{2}\right){r_{i}}^{2}{r_{e}}^{2}\\ \;+\;4{\left(\ln\left(\frac{r_{e}}{r_{i}}\right)\right)}^{2}\left({r_{e}}^{2}+{r_{i}}^{2}\right){r_{i}}^{2}{r_{e}}^{2} \end{array}} \end{array}$$

This equation highlights the geometrical difference between the hollow cylinder and the planar configuration. In the planar configuration, the geometry is determined by a single factor: thickness. However, the geometry of the hollow cylinder actuator depends on two independent geometrical factors: either two radii or one radius and a thickness.

The electrostatic pressure (*P*) on the internal and external electrodes, respectively, are given by:

(25) $$P_{r_{i}}=\frac{F}{A_{r_{i}}}=\frac{\left|\nabla U\right|}{2\pi r_{i}l}\;$$

(26) $$P_{r_{e}}=\frac{F}{A_{r_{e}}}=\frac{\left|\nabla U\right|}{2\pi r_{e}l}$$

By substituting the respective values from Equation 24 into the two above equations, the electrostatic pressure at the internal and external radii can be calculated as follows:

(27) $$\begin{array}{ccc} P_{r_{i}} & = & \frac{\varepsilon \Delta V^{2}}{2{\left(\ln\left(\frac{r_{e}}{r_{i}}\right)\right)}^{2}{r_{i}}^{2}r_{e}\left({r_{e}}^{2}-{r_{i}}^{2}\right)}\\ & & \times \sqrt{\begin{array}{c} {r_{i}}^{6}+{r_{e}}^{6}-{r_{i}}^{2}{r_{e}}^{4}-{r_{e}}^{2}{r_{i}}^{4}+8\ln\left(\frac{r_{e}}{r_{i}}\right)\left({r_{e}}^{2}-{r_{i}}^{2}\right){r_{i}}^{2}{r_{e}}^{2}\\ \;+4{\left(\ln\left(\frac{r_{e}}{r_{i}}\right)\right)}^{2}\left({r_{e}}^{2}+{r_{i}}^{2}\right){r_{i}}^{2}{r_{e}}^{2} \end{array}} \end{array}$$

(28) $$\begin{array}{ccc} P_{r_{e}} & = & \frac{\varepsilon \Delta V^{2}}{2{\left(\ln\left(\frac{r_{e}}{r_{i}}\right)\right)}^{2}r_{i}{r_{e}}^{2}\left({r_{e}}^{2}-{r_{i}}^{2}\right)}\\ & & \times \sqrt{\begin{array}{c} {r_{i}}^{6}+{r_{e}}^{6}-{r_{i}}^{2}{r_{e}}^{4}-{r_{e}}^{2}{r_{i}}^{4}+8\ln\left(\frac{r_{e}}{r_{i}}\right)\left({r_{e}}^{2}-{r_{i}}^{2}\right){r_{i}}^{2}{r_{e}}^{2}\\ \;+4{\left(\ln\left(\frac{r_{e}}{r_{i}}\right)\right)}^{2}\left({r_{e}}^{2}+{r_{i}}^{2}\right){r_{i}}^{2}{r_{e}}^{2} \end{array}} \end{array}$$

In the following, we connect the electrostatic pressures $P_{r_{i}}$ and $P_{r_{e}}$ to the mechanical strains induced by the given pressures. So far, the HFDEA is considered to be in the non‐actuated state, and the electrostatic characteristic is defined based on the generic internal radius (*r_i_*), external radius (*r_e_*) and electrode length (*l*).

Similar to the planar configuration, where the thickness in the actuated state is approximated by its initial value for strain calculations, here, for calculating the exerted electrostatic pressure and subsequently the actuation strain, the internal and external radius values (and therefore the thickness, (*r_e_* − *r_i_*) at the actuated state are approximated by their initial values. This simplification avoids the complexities associated with accounting for dynamic geometric changes during actuation. However, it assumes small strain conditions (≤10%), ensuring that the derived equations remain valid for such deformations.^[^ ^41^ ^]^ Beyond this range, the linearization and thickness approximations may lead to inaccuracies. The pressures on the actuator's internal and external surfaces immediately result in the elastomer's expansion in the z‐direction. To simplify the analytical solution in the small strain range, we assume the elastomer is linearly elastic (governed by Hooke's Law), isotropic, and homogeneous.

Electrostatic pressures applied to the elastomer create mechanical stresses that result in displacement (strain) of the elastomer. To determine the resulting strains, it is necessary first to identify the associated displacement fields. In cylindrical geometry, the equations of motion for linear elastic materials are referred to as the Navier‐Lamé equations. The equations simplify once applied to the cylindrical coordinates of the Navier‐Stokes equations. They apply Hooke's law to relate stress and strain and are specially arranged in cylindrical coordinates to simplify the analysis of the displacement fields.

The Navier‐Lamé equations, also known as the Navier‐Cauchy equations, are applied in the equilibrium state to calculate the strains. The stationary solutions are used to capture the final actuation state without accounting for their dynamic behavior. The displacement components in the 𝑟, 𝜃, and 𝑧 directions are expressed as *u_r_*, *u*_θ_, and *u*_z_, respectively.^[^ ^42^ ^]^

The Navier‐Lamé equation can be written in vector format as:

(29) $$\mu \nabla^{2}u+\left(\lambda +\mu \right)\nabla \left(\nabla \cdot u\right)+b=\rho \;\frac{\partial^{2}u}{\partial t^{2}}=0$$

where *b* is the body force, which, due to no pre‐stretch assumption, is set to zero, and ρ is the elastomer density. Also, due to the stationary assumption, the term $\frac{\partial^{2}u}{\partial t^{2}}$ is set to zero. *λ* and *µ* are Lamè’s constants used in the Navier‐Lamé equations in cylindrical coordinates and defined as:

(30) $$\lambda =\frac{\nu Y}{\left(1+\nu \right)\left(1-2\nu \right)}\;$$

(31) $$\mu =\frac{Y}{2(1+\nu )}\;$$

where *Y* is the Young's modulus and ν is the Poisson's ratio of the dielectric elastomer. The Poisson's ratio is set to 0.499 in all derivations within this work due to approximate incompressibility while avoiding singularities in the mathematical calculations.^[^ ^43^ ^]^ The Navier‐Lamé equation (Equation 29) for each direction can be expressed as follows:

The radial equation is given by:

(32) $$\begin{array}{ccc} & & \lambda \left(\frac{1}{r}\frac{\partial u_{r}}{\partial r}+\frac{1}{r}\frac{\partial u_{\theta }}{\partial \theta }+\frac{\partial u_{z}}{\partial z}\right)+\left(\lambda +2\mu \right)\\ & & \;\times \left(\frac{\partial^{2}u_{r}}{\partial r^{2}}+\frac{1}{r}\frac{\partial u_{r}}{\partial r}-\frac{u_{r}}{r^{2}}+\frac{1}{r^{2}}\frac{\partial^{2}u_{r}}{\partial \theta^{2}}+\frac{\partial^{2}u_{r}}{\partial z^{2}}\right)=0 \end{array}$$

The angular equation can likewise be expressed as:

(33) $$\begin{array}{ccc} & & \lambda \left(\frac{1}{r}\frac{\partial u_{r}}{\partial r}+\frac{1}{r}\frac{\partial u_{\theta }}{\partial \theta }+\frac{\partial u_{z}}{\partial z}\right)+\left(\lambda +2\mu \right)\\ & & \;\times \left(\frac{\partial^{2}u_{\theta }}{\partial r^{2}}+\frac{1}{r}\frac{\partial u_{\theta }}{\partial r}-\frac{u_{\theta }}{r^{2}}+\frac{1}{r^{2}}\frac{\partial^{2}u_{\theta }}{\partial \theta^{2}}+\frac{\partial^{2}u_{\theta }}{\partial z^{2}}\right)=0 \end{array}$$

and finally, the axial equation is:

(34) $$\begin{array}{ccc} & & \lambda \left(\frac{1}{r}\frac{\partial u_{r}}{\partial r}+\frac{1}{r}\frac{\partial u_{\theta }}{\partial \theta }+\frac{\partial u_{z}}{\partial z}\right)+\left(\lambda +2\mu \right)\\ & & \;\times \;\left(\frac{\partial^{2}u_{z}}{\partial r^{2}}+\frac{1}{r}\frac{\partial u_{z}}{\partial r}+\frac{1}{r^{2}}\frac{\partial^{2}u_{z}}{\partial \theta^{2}}+\frac{\partial^{2}u_{z}}{\partial z^{2}}\right)=0 \end{array}$$

By assuming no movement in the θ direction (*u*_θ_ =  0) and considering the radial displacement to depend only on radial coordinates (*u_r_* = *u_r_* (*r*)), and the axial displacement depending only on the axial coordinates (*u_z_* = *u_z_* (*z*)), the radial and axial equations can be simplified to:

(35) $$\frac{d}{dr}\;\left(\frac{1}{r}\frac{d(ru_{r)}}{dr}\right)=0$$

(36) $$\frac{\partial^{2}u_{z}}{\partial z^{2}}=0$$

The general solutions for the two equations are:

(37) $$u_{r}=\frac{C_{1}r}{2}+\frac{C_{2}}{r}$$

(38) $$u_{z}=C_{3}\;z+C_{4}$$

where C_1_,C_2_,C_3_, *and* C_4_ are constants.

In the cylindrical coordinate system, the relationships between radial strain component (*S_r_)*, circumferential strain component (*S*_θ_), axial strain component (*S_z_*), and the corresponding displacements are defined as follows:

(39) $$S_{r}=\frac{du_{r}}{\mathrm{dr}}\;$$

(40) $$S_{\theta }=\frac{u_{r}}{r}+\frac{l}{r}\frac{du_{\theta }}{d\theta }$$

(41) $$S_{z}=\frac{du_{z}}{\mathrm{dz}}\;$$

The stresses in cylindrical coordinates are radial stress (*T_rr_*), circumferential stress (*T*_θθ_), and axial stress (*T_zz_*). The relationship between the stresses, with strains derived using Hooke's law, can be expressed as Lamé constants:

(42) $$T_{rr}=\lambda (S_{r}+S_{\theta }+S_{z})+2\mu S_{r}$$

(43) $$T_{\theta \theta }=\lambda (S_{r}+S_{\theta }+S_{z})+2\mu S_{\theta }$$

(44) $$T_{zz}=\lambda (S_{r}+S_{\theta }+S_{z})+2\mu S_{z}$$

Here, stress in the z direction is assumed to be zero (*T_zz_* =  0). Additionally, the radial stresses at the internal and external boundaries are equal to the electrostatic pressures ($T_{rr}{|}_{r_{i}}=P_{r_{i}}$, $T_{rr}{|}_{r_{e}}=-P_{r_{e}}$). By applying these boundary conditions to Equations (37)–(44), the stresses can be expressed as:

(45) $$T_{rr}=-\frac{P_{r_{e}}{r_{e}}^{2}-P_{r_{i}}{r_{i}}^{2}}{{r_{e}}^{2}-{r_{i}}^{2}}+\frac{\left(P_{r_{e}}-P_{r_{i}}\right){r_{i}}^{2}{r_{e}}^{2}}{{r_{e}}^{2}-{r_{i}}^{2}}\frac{1}{r^{2}}$$

(46) $$T_{\theta \theta }=-\frac{P_{r_{e}}{r_{e}}^{2}-P_{r_{i}}{r_{i}}^{2}}{{r_{e}}^{2}-{r_{i}}^{2}}-\frac{\left(P_{r_{e}}-P_{r_{i}}\right){r_{i}}^{2}{r_{e}}^{2}}{{r_{e}}^{2}-{r_{i}}^{2}}\frac{1}{r^{2}}$$

(47) $$T_{zz}=0$$

The strain components (*S*) are expressed as:

(48) $$S_{r}=\frac{1}{Y}\;[T_{rr}-\nu (T_{\theta \theta }+T_{zz})]$$

(49) $$S_{\theta }=\frac{1}{Y}\;[T_{\theta \theta }-\nu (T_{rr}+T_{zz})]$$

(50) $$S_{z}=\frac{1}{Y}\;[T_{zz}-\nu (T_{rr}+T_{\theta \theta })]$$

Equations (48)–(50) enable the calculation of the total axial stain, as well as the internal and external radial strains, expressed in terms of engineering strain. These relationships can be formulated as follows:

(51) $$s_{z}=\frac{\Delta l}{l_{0}}=\frac{1}{l_{0}}\;\int_{0}^{l}S_{z}\mathrm{dz}$$

(52) $$\frac{\Delta r_{i}}{r_{i}}=\frac{1}{2\pi }\;\int_{0}^{2\pi }S_{\theta }(r_{i})\;d\theta$$

(53) $$\frac{\Delta r_{e}}{r_{e}}=\frac{1}{2\pi }\;\int_{0}^{2\pi }S_{\theta }(r_{e})\;d\theta$$

By substituting the stress expressions from Equations (45)–(47) into the strain equation for the z direction (Equation 50) and taking the integral, as expressed in Equation (51), the total axial strain can be determined as:

(54) $$s_{z}=\frac{\Delta l}{l_{0}}=\frac{\left(P_{r_{e}}{r_{e}}^{2}-P_{r_{i}}{r_{i}}^{2}\right)}{Y\left({r_{e}}^{2}-{r_{i}}^{2}\right)}\;$$

To simplify the axial strain, after inserting Equations (27) and (28) in Equation (54), the strain equation is expressed as:

(55) $$\begin{array}{ccc} s_{z} & = & \frac{\varepsilon }{Y{\left(r_{e}-r_{i}\right)}^{2}}\;\frac{\Delta V^{2}}{2{\left(\ln\left(\frac{r_{e}}{r_{i}}\right)\right)}^{2}r_{i}r_{e}\left(r_{i}+r_{e}\right)}\\ & & \times \sqrt{\begin{array}{c} {r_{i}}^{6}+{r_{e}}^{6}-{r_{i}}^{2}{r_{e}}^{4}-{r_{e}}^{2}{r_{i}}^{4}+8\ln\left(\frac{r_{e}}{r_{i}}\right)\left({r_{e}}^{2}-{r_{i}}^{2}\right){r_{i}}^{2}{r_{e}}^{2}\\ \;+4{\left(\ln\left(\frac{r_{e}}{r_{i}}\right)\right)}^{2}\left({r_{e}}^{2}+{r_{i}}^{2}\right){r_{i}}^{2}{r_{e}}^{2} \end{array}} \end{array}$$

Equation (55) represents the conclusive form of the strain equation in fiber format, incorporating both material and geometric factors, and it is essential for describing the electromechanical behavior of the HFDEAs. The strain equation in fiber format (Equation 55) can be presented in a similar style to the strain equation in planar format (Equation 4) by introducing two parameters, one to account for the elastomer properties (𝛼) and the other to account for the geometric factors (𝛽).

The parameter 𝛼 represents the actuator's electro‐mechanical sensitivity, expressed as:

(56) $$\alpha =\frac{\varepsilon }{Y}\;$$

This is a standard measure to evaluate dielectric elastomer performance since the ratio represents the electrical storage capacity over the mechanical resistance to deformation of the elastomer.^[^ ^44^ ^]^

Another parameter, β, is introduced to reflect the influence of the actuator's cylindrical design on the strain and is defined as:

(57) $$\begin{array}{ccc} \beta & = & \frac{1}{{\left(r_{e}-r_{i}\right)}^{2}}\;\frac{1}{2{\left(\ln\left(\frac{r_{e}}{r_{i}}\right)\right)}^{2}r_{i}r_{e}\left(r_{i}+r_{e}\right)}\\ & & \times \sqrt{\begin{array}{c} {r_{i}}^{6}+{r_{e}}^{6}-{r_{i}}^{2}{r_{e}}^{4}-{r_{e}}^{2}{r_{i}}^{4}+8\ln\left(\frac{r_{e}}{r_{i}}\right)\left({r_{e}}^{2}-{r_{i}}^{2}\right){r_{i}}^{2}{r_{e}}^{2}\\ \;+4{\left(\ln\left(\frac{r_{e}}{r_{i}}\right)\right)}^{2}\left({r_{e}}^{2}+{r_{i}}^{2}\right){r_{i}}^{2}{r_{e}}^{2} \end{array}} \end{array}$$

The validity of 𝛽 is limited to small strain conditions (<10%), for which the strains can be calculated directly from the initial geometrical dimensions (*r_i_*, *r_e_*). It is important to note that this assumption is influenced not only by β but also by α. However, in cases where fiber has an extremely thin wall or a very large radius ratio (*k = r_e_/r_i_*), as will be discussed in section 3.1, *β* can take excessively large values, potentially leading to strains that exceed the small strain assumption. Therefore, the formulation is not generally applicable to such geometries. To express the strain in a form comparable to the strain equation in convectional configurations (such as Equation 4), where the dependency on the electric field is explicitly highlighted, β can be structured as:

(58) $$\beta =\frac{\beta_{0}}{{\left(r_{e}-r_{i}\right)}^{2}}\;$$

where β_0_ is introduced for convenience, as it simplifies the referral to the geometrical contribution in the subsequent equation, involving the electric field as derived in Equation (62). It is defined as:

(59) $$\begin{array}{ccc} \beta_{0} & = & \frac{1}{2{\left(\ln\left(\frac{r_{e}}{r_{i}}\right)\right)}^{2}r_{i}r_{e}\left(r_{i}+r_{e}\right)}\\ & & \times \sqrt{\begin{array}{c} {r_{i}}^{6}+{r_{e}}^{6}-{r_{i}}^{2}{r_{e}}^{4}-{r_{e}}^{2}{r_{i}}^{4}+8\ln\left(\frac{r_{e}}{r_{i}}\right)\left({r_{e}}^{2}-{r_{i}}^{2}\right){r_{i}}^{2}{r_{e}}^{2}\\ \;+4{\left(\ln\left(\frac{r_{e}}{r_{i}}\right)\right)}^{2}\left({r_{e}}^{2}+{r_{i}}^{2}\right){r_{i}}^{2}{r_{e}}^{2} \end{array}} \end{array}$$

With the introduction of the definitions of the three parameters, 𝛼, 𝛽, and 𝛽_o_, the actuation strain equation for fiber actuators can be expressed as:

(60) $$s_{z,fiber}=\alpha \beta \Delta V^{2}$$

To further refine the expression of strain in the fiber actuator configuration, we can express it in terms of the average electric field across the cylindrical geometry:

(61) $$E_{avg}=\frac{\Delta V}{\left(r_{e}-r_{i}\right)}\;$$

By substituting the average electric field expression into Equation (60), the strain becomes:

(62) $$s_{z,fiber}=\alpha \beta_{0}{E_{avg}}^{2}$$

The axial strain is, as expected, directly related to the square of the applied voltage (Δ𝑉), with prefactors 𝛼 and 𝛽 taking into account the material properties and geometry. Maximization of the strain performance of the actuator can thus be conducted by optimizing both the material properties and the geometrical configuration to suit specific application needs. The presentation of the axial strain of fiber actuators in this style (Equation (62)) further facilitates the comparison between the strain in the planar format versus the hollow fiber format actuator configurations, as will be conducted in section 3.1.

### 2.2 Numerical Modeling of HFDEAs

**Figure 3:**
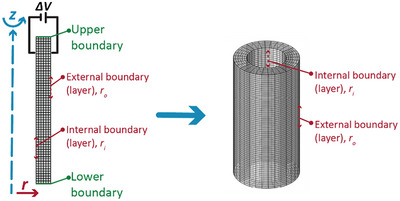
Illustration of the modeling process: the 2D axisymmetric meshed design of the cylindrical geometry used for simulations (left) is solved in the 2D framework. The resulting geometry is then revolved to visualize the corresponding three‐dimensional representation of the hollow cylinder (right).

The assumptions of linear elasticity and the absence of pre‐stretching lead to predictions that do not fully resemble the operational conditions and nature of the dielectric elastomer actuators. The other mathematical simplifications, such as the assumption of uniform radial expansion at the fiber's end and relating the electrostatic pressure to the initial geometrical properties, also contribute to reduced accuracy in predicting the actuator's deformation behavior. Linear elasticity fails to cover the mechanical response under pre‐stretched conditions because the pre‐stretch leads to overall larger deformations than what lies within the linear envelope of the elastomer. Therefore, more advanced models are needed to validate the result of the analytical approach and better investigate the HFDEA's behavior. We employ COMSOL Multiphysics to simulate the mechanical and electrical behaviors of dielectric elastomer actuators (DEAs). The geometry is considered axisymmetric, and the physics of the HFDEA is defined in COMSOL using the modules: a) solid mechanics to simulate elastomer mechanical behavior and deformations, b) electrostatics to model the electric field distribution, and c) electromechanical forces for multiphysics coupling to capture electro‐mechanical interactions. The mechanical properties are described using a hyperelastic material model with parameters determined from experimental data. The empirical, two‐parameter Mooney‐Rivlin model fits the experimental data well (see Section S2, Supporting Information). Mechanical and electrical characterizations are performed before the simulations to feed the model. The mesh information and simulation setup are shown in **Figure** **3**.

### 2.3 Fabrication and Characterization

Two different sizes of hollow fibers were fabricated from polydimethylsiloxane (PDMS) elastomers to validate the model predictions. Thiol‐ene click chemistry was adopted via a wet spinning process using a UV lamp to cure the elastomers, and actuator assembly was conducted using a method established by Kang et al.^[^ ^14^ ^]^ The materials for manufacturing silicone elastomer fibers were as follows: [4%–6% mercaptopropyl]methylsiloxane‐dimethylsiloxane copolymer (SMS‐042, molecular weight = 6869 g mol^−1^) and vinyl‐terminated polydimethylsiloxane (DMS‐V31, molecular weight = 28000 g mol^−1^), both purchased from Gelest Inc., USA. Chloroform, 2,2‐dimethoxy‐2‐phenylacetophenone (DMPA), 1‐ethyl‐3‐methylimidazolium bis(trifluoromethylsulfonyl)imide ([Emim][TFSI]), ethanol, and acetone were acquired from VWR Chemicals BDH. Polyvinyl acetate (PVAc, molecular weight = 90000 g mol^−1^) was obtained from Polysciences Inc., USA.

The fibers were produced by combining DMS‐V31 and SMS‐042 via thiol‐ene click chemistry, with DMPA as the photo‐initiator. The fibers were formed via a wet spinning procedure involving a dual‐needle coaxial spinneret positioned inside a 3 mm deep ethanol bath. The spinneret enabled the precise injection of silicone and ethanol solutions, which were promptly cured using UV light. By utilizing this configuration, it was possible to modify the fabrication parameters, including the duration of UV exposure, spinning speed, and the proportion between the internal solvent and the outside silicone melt. Consequently, this facilitated the creation of hollow fibers with adjustable wall thicknesses.

**Figure 4:**
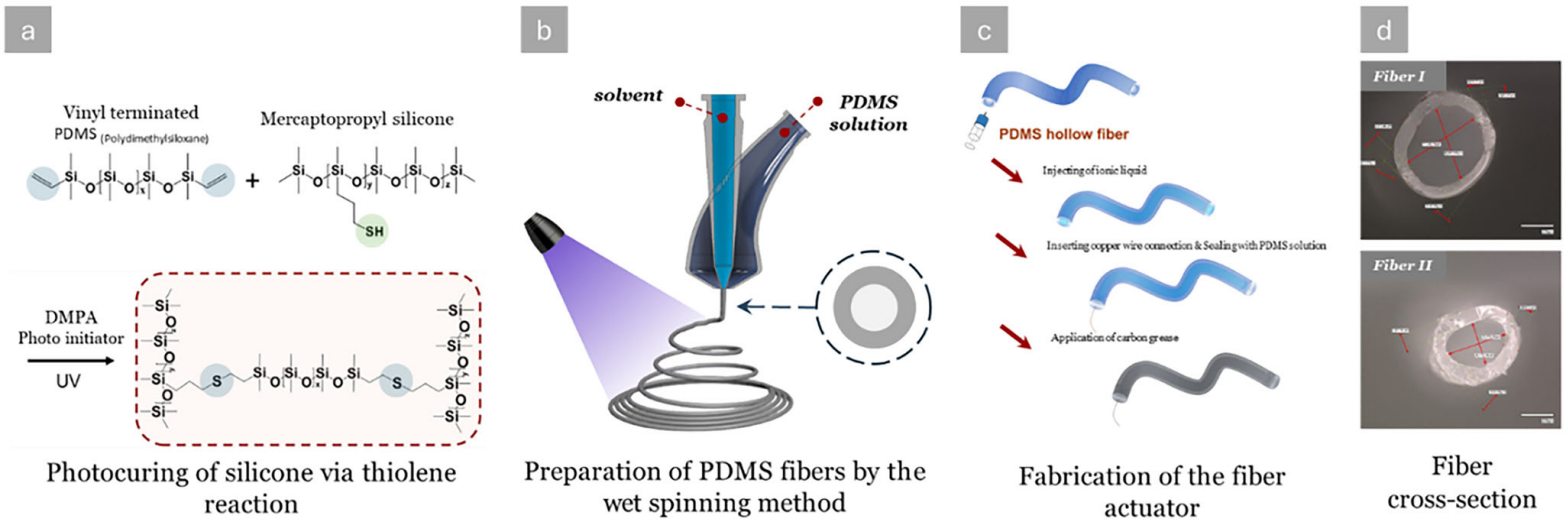
a) Crosslinking reaction for the silicone preparation, b) The setup for the wet spinning process of hollow fiber elastomers, and c) The assembly steps, including the injection of the internal electrode and application of the external electrode, d) microscopic image of a cross‐section of fabricated fibers.

The actuators were prepared by injecting the hollow fibers with ionic liquid to function as the internal electrode. A slender copper wire connected this internal electrode and an external high‐voltage source. The fiber ends were sealed using the same silicone mixture and cured using UV light to achieve firm and secure sealing. The last stage of the manufacturing process entailed applying carbon grease to the outside surface of the fibers to establish it as the conductive outer electrode. A schematic overview of the materials, fabrication steps, and a representative fiber cross‐section is provided in **Figure** **4**. The geometric parameters of the produced fibers are summarized in **Table** **1**.

**Table 1:** The prepared silicone elastomer fibers and their geometries.

Samples	Outer Diameter [µm]	Inner Diameter [µm]	Wall Thickness [µm]
Fiber I	469	283	105
Fiber II	605	443	161

The size of fibers was determined using an optical microscope (Leica DMLS, Germany). Dielectric Relaxation Spectroscopy (DRS) analysis was conducted using a high‐performance Novocontrol Alpha‐A frequency analyzer (Novocontrol Technologies GmbH & Co, Germany), applying an electric field of ≈1 V mm^−1^ across a frequency spectrum of 10^−1^ to 10^6^ Hz, at ambient temperature. Copper sheets were used as electrodes, and the samples had a diameter of 20 mm and a thickness of 1 mm.

Instron 3340 system (Instron, US) was used to measure tensile properties. Samples were stretched at a rate of 10 mm min^−1^ from an initial active elastomer length of 10 mm. The tensile data and the fitted Mooney‐Rivlin model are shown in Section S2 (Supporting Information).

Actuation was conducted with a Petapico‐Voltron high‐voltage power supply. Each actuation was subjected to an increasing voltage increment from 250 V to 2 kV. The changes in fiber length were captured with a LUMIX DMC‐G80 digital camera and analyzed using Tracker software for video analysis. The experiments were performed in triplicates for each fiber size.

## 3 Results and Discussion

**Figure 5:**
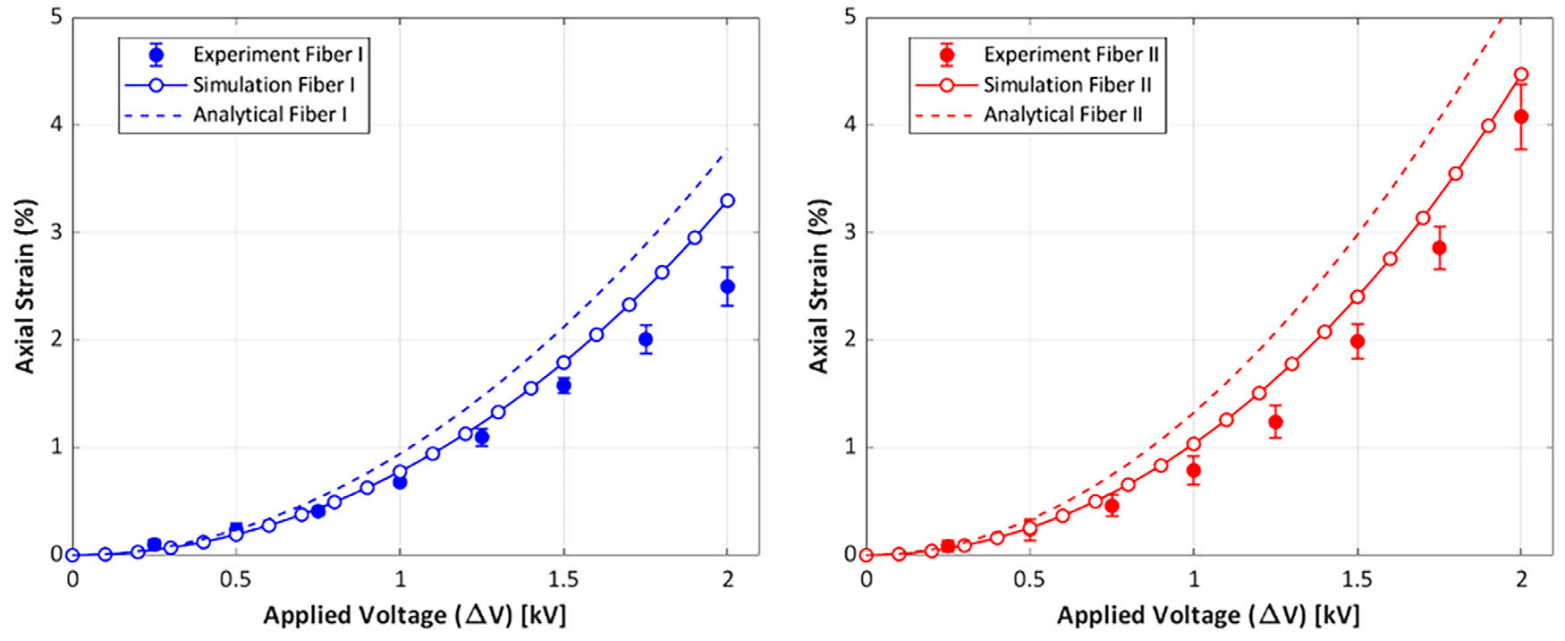
Comparison of analytical method, simulation results and experimental data of axial strain as a function of the applied electrical voltage for two different fiber geometries.

The material properties used for the numerical and analytical predictions were obtained through mechanical and electrical testing, and the predicted values were subsequently validated against experimental actuation test results. This comparison is illustrated in **Figure** **5**, which shows the axial strain determined in three ways: 1) experimental data from the actuation test, 2) the simulation results from the numerical modeling, and 3) the analytical predictions. This comparison includes two different HFDEA geometries: fiber I (blue) and fiber II (red).

The experimental data exhibits a quadratic increase in the axial strain as the applied voltage increases. This trend aligns well with both analytical and numerical predictions for the electrostatic strains, as derived from Equation (62), as well as the original theories for the actuation of planar geometries.

However, both theoretical and numerical values are higher than experimental data. This difference can be attributed to unaccounted factors such as electrical losses in the electrodes due to their nonideal conduction and dielectric and mechanical losses within the elastomer. Compared to the analytical model, the numerical method incorporates more realistic boundary conditions. Specifically, in the numerical method, the fiber ends are restricted from radial expansion due to the experimental connection constraints. In contrast, the analytical model assumes that the fiber experiences a uniform and unrestricted radial expansion along its entire length, including the ends. Additionally, the numerical approach also captures the nonlinear mechanical properties of the actuator, as a result of nonlinear strain stress and the influence of geometric boundary conditions at the constrained ends, amongst others. These effects become more pronounced at more significant deformations but are not accounted for in the analytical equation, which assumes linear elasticity and small‐strain conditions. These considerations result in strain estimates from the numerical model that are closer to the experimental values than the prediction of the analytical equation.

With the verified numerical model, we can perform further comparisons. For example, the model allows us to explore the strain differences between HFDEAs and planar films, providing insights into strain performance for different geometries. Additionally, the model enables the study of difficult‐to‐measure values, such as the radial strain of small‐sized HFDEAs, which are challenging to measure experimentally. Finally, the design of the HFDEAs can be optimized for strain, along with another essential performance indicator, namely the holding force, by use of the numerical model.

### 3.1 Comparison Between Fiber and Film, Analytical Approach

To analytically compare the actuation strain of HFDEA against the actuation strain in planar format, we evaluate the axial strain of the fiber and the in‐plane expansion strain of the planar film with identical thicknesses. This choice of strains allows us to compare the axial strain in fiber to a similar strain in planar format, both being perpendicular to the electric field, by using Equations (4) and (62). We set the thickness of the planar format (*th*) to be the same as the thickness of hollow fiber, namely *r_e_* − *r_i_*, as well as equaling the average field in the fiber to that of the planar configuration. Thereby we reach the following expression for the strain ratio:

(63) $$\frac{s_{z,fiber}}{s_{x,film}}=2\beta_{0}$$

**Figure 6:**
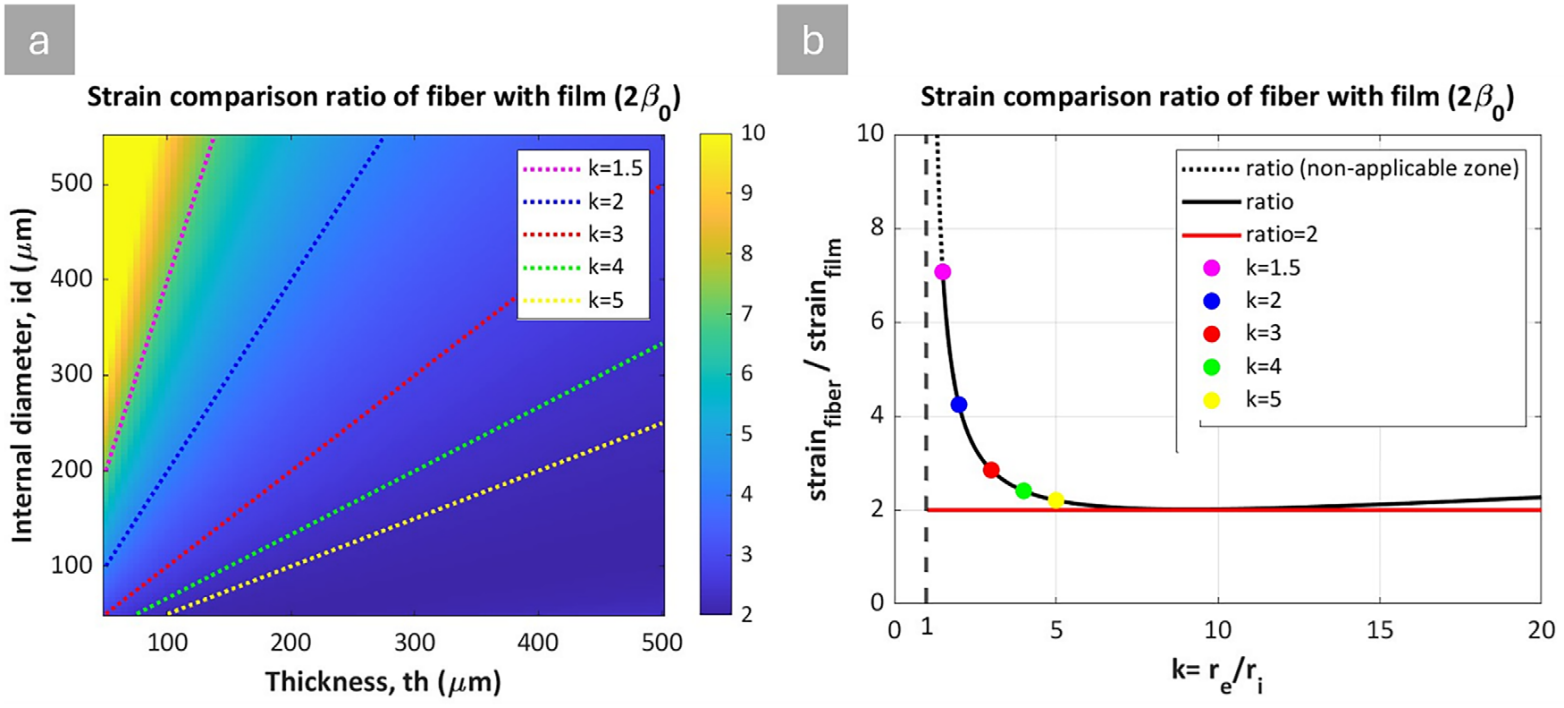
Analytical solution predictions: strain comparisons. a) Heatmap showing the strain ratio of HFDEAs compared to planar film DEAs (equation 63) as a function of fiber thickness and internal diameter. The working line corresponds to different radius ratios (k = r_e_/r_i_), where 𝑟_𝑒_ and 𝑟_𝑖_ are the outer and inner radii, and is indicated by dashed lines. b) Strain ratios between HFDEAs and planar film DEAs plotted against radius ratio (*k*), with strain limited to ≤10%. Markers in (b) correspond to the working lines in the heatmap in (a).

In **Figure** **6**a, the strain ratio is plotted as a function of the thickness and internal diameter of the fiber. The results show that HFDEAs achieve greater strain than the corresponding planar film with the same thickness for all parameters. Figure 6b provides a clearer view, showing the strain ratio between the fiber and film as a function of the radius ratio, defined as $k=\frac{r_{e}}{r_{i}}\;$. As the radius ratio increases from *k *= 1, the strain ratio decreases rapidly to a value of two, where it, for a broad range of k, remains relatively constant. The upturn for *k* approaching 1 is due to the infinitely thin elastomer film. Approaching k = 1, the DEAs exhibit high strains that fall outside the eligibility of the current analytical approach, designed to capture low‐strain behaviors (up to ≈10% due to the simplified equations used). The plot has, therefore, been cut off when strains became excessive. To explain the rapid increase toward k = 1, we need to look into the possible 3D strains of the fiber geometry. The fiber can deform in three dimensions, namely in circumference, thickness, and length. However, if deformed purely elastically in a tensile test, one would observe only changes in length and thickness due to the absence of a driving force for increased circumference. For the planar geometry, the film will expand in both the plane of the film and in thickness, i.e., in three dimensions. However, in actuation, there is a driving force for the circumference expansion of the fiber due to the electrical charges on the electrodes, but this electrical force is opposed by the mechanical force. In other words, for the planar geometry, the decrease in thickness leads to actuation in two dimensions. In contrast, for the fiber geometry, the decreased thickness leads to deformation in mainly one direction (length).

As 𝑘 continues to increase, the strain ratio is again increasing. This time, the increase is a result of the different charge densities on the two electrodes. This is further discussed in Section 3.2.

**Figure 7:**
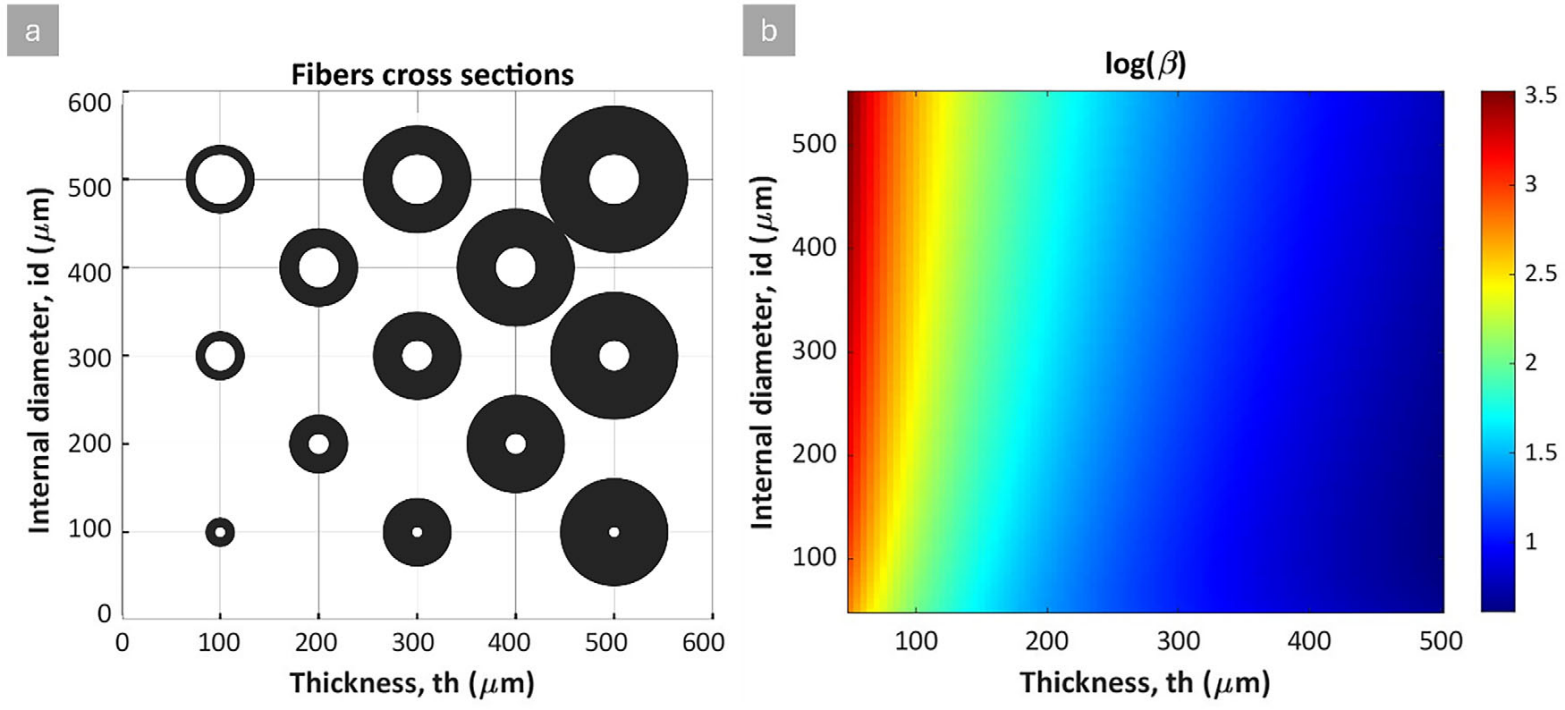
Analytical method visualization: a) Illustration of cross‐sections of fibers with different geometries. b) Heatmap illustrates the values of log(𝛽) across varying thicknesses (𝑡ℎ) and internal diameters (𝑖𝑑) of the fibers.

In addition to the strain comparison, the parameter β introduced earlier (Equation (57)) encapsulates the combined effects of internal diameter (id) and wall thickness (th) on actuator behavior. **Figure** **7a** illustrates the cross‐sections of fibers based on varying internal diameters and thicknesses, providing a visualization for understanding the effect of geometrical parameters. Figure 7b maps the parameter 𝛽 as a function of the internal diameter (𝑖𝑑) and thickness (𝑡ℎ) of the fibers, corresponding to the geometries on the left side of the figure. Regions with higher 𝛽 values (shown in warmer colors) correspond to fibers with larger internal diameters and thinner walls. These configurations allow for increased actuation due to the low thickness. Even slight increases in wall thickness, particularly from very thin membranes, cause a rapid drop in actuation performance, emphasizing that wall thickness has a stronger influence on relative actuator performance than the internal diameter, as expected.

Summarizing, the analysis indicates that to enhance the performance of HFDEAs to achieve greater strains, it requires prioritizing thinner walls and bigger internal diameters in the design, in other words, approaching a cylindrical geometry with small curvature.

However, this comparison does not account for the mass of the material required for a given strain, an important factor to consider. This aspect is explored further in Section 3.3 through mass‐normalized strain and force evaluations.

### 3.2 Radial Strain

Measuring the radial strain of fibers, particularly those with small diameters, during actuation and operation is a challenging task. However, with the validated numerical model based on actuation strain comparison, we can estimate this property reliably. By analytically comparing the electrostatic pressures exerted on the internal and external layers of fiber, as denoted in Equations (27) and (28), the relationship between the electrostatic pressures can be expressed as:

(64) $$\frac{P_{r_{i}}}{P_{r_{e}}}=\frac{r_{e}}{r_{i}}\;$$

This expression is always greater than 1, as the external radius is larger than the internal radius. It indicates that the internal layer consistently experiences greater outward pressure compared to the inward pressure on the external boundary. This pressure difference arises due to the higher surface charge density on the internal layer compared to the external layer. The unlike charges on the two layers and the electrostatic attraction between them might initially suggest that the layers would move toward each other (causing outward movement of the internal layer and inward movement of the external layer) while the fiber extends. However, the absolute strain of the external layer is directed outward. This occurs because the higher surface charge density on the internal layer exerts a stronger outward force on the fiber walls, ultimately resulting in an increase in the external diameter to minimize the surface charge density of, particularly, the inner surface. For low values of *k*, where the inner and outer diameters approach each other, this difference in charge density becomes insignificant.

**Figure 8:**
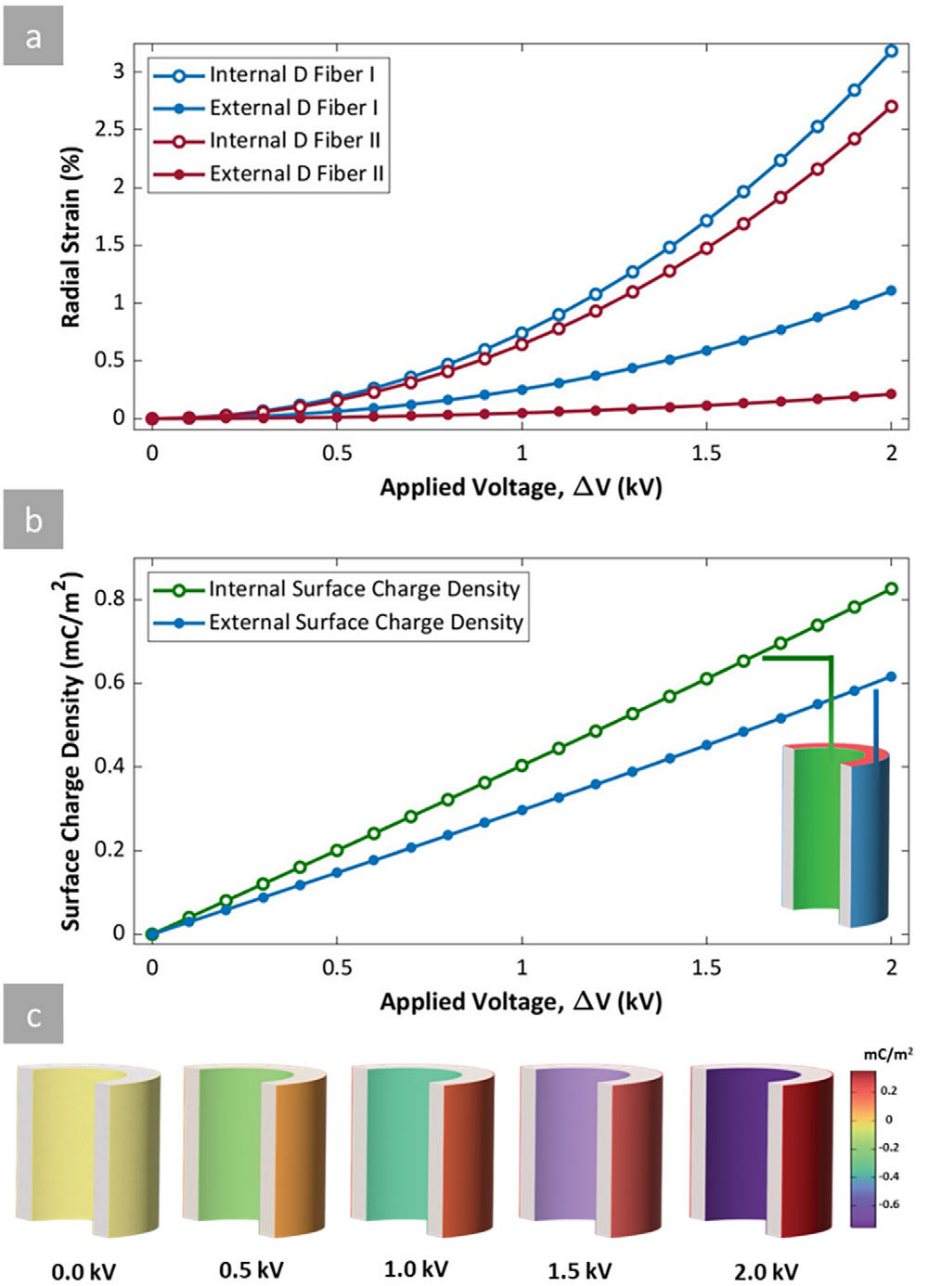
Simulation results: a) A comparison of the radial strains of the internal and external surfaces at different voltages in HFDEAs. b) A comparison of surface charge density (Fiber II) on internal versus external layers of HFDEAs showing the higher charge density on the internal layer. c) Visualization of surface charge density distribution in HFDEAs (Fiber II) through a longitudinal cut of the hollow fiber, exposing both internal and external layers and illustrating charge density variations under different applied voltages.

The numerical results demonstrate how this pressure difference influences the strain distribution across the fiber. **Figure** **8a** illustrates the radial strain of both inner and outer layers under varying applied voltages over the two types of fibers, showing that the internal layer experiences greater relative radial expansion than the external layer in HFDEAs. This increased expansion of the outer radius is due to the higher surface charge density on the internal layer, as shown in Figure 8b,c.

The observed cylindrical expansion as a result of the internal strain has significant implications for the HFDEA design, particularly when using ionic liquid as the internal electrode. During actuation, both the axial strain and the internal radius expansion act to increase the internal volume of the fiber. To ensure proper operation of the fiber actuator, it is necessary to account for the additional volume of liquid required. In our experiments, this requirement was addressed by having the first centimeter of the elastomer free of external electrode coverage and ensuring the internal connection wire extended adequately into the active region. That inactive area helps accommodate some of the extra liquid volume needed for effective actuation. However, for large actuation or long actuators, a different internal electrode is required.

**Figure 9:**
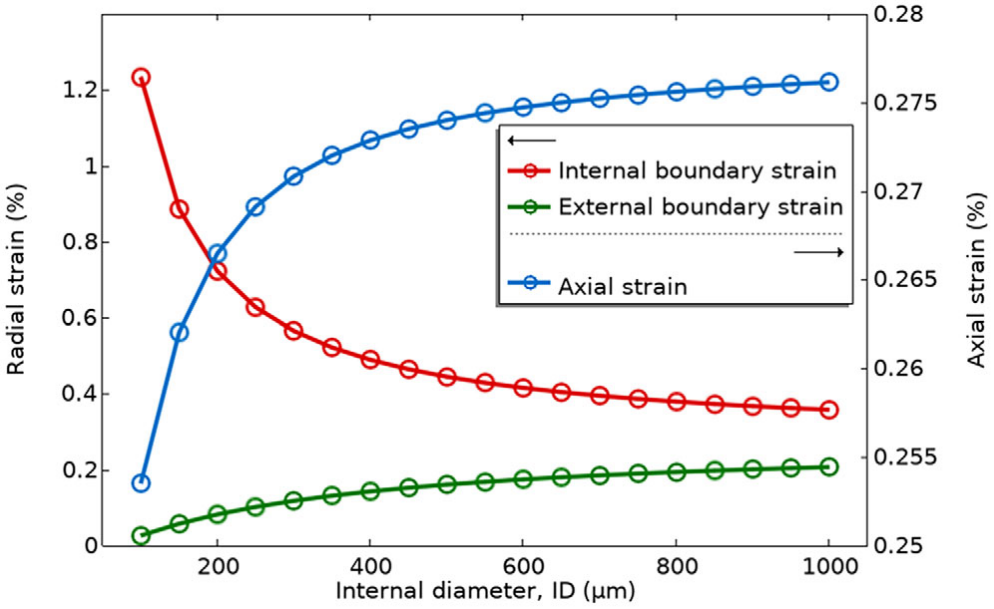
Simulation results of the impact of internal diameter on the actuation strains of HFDEAs at 1 kV applied voltage with a fixed initial wall thickness (100 µm). The following strains are shown: The axial strain (blue) internal boundary strain (red), and the external boundary strain (green).

For similar thicknesses of HFDEAs, the internal diameter affects the axial strain as well as internal and external radial strains. **Figure** **9** demonstrates this influence under a constant applied voltage of 1 kV and a wall thickness of 100 µm. Here, we observe that the axial strain (blue line) increased with internal diameter, as the surface charge densities of the two surfaces approach each other to give a constant actuation strain above a certain internal diameter. The red line shows the reduction in the radial strain as the internal diameter increases, which reduces the difference in surface charge density and thereby strongly affects the radial strain of the internal surface for small internal diameters. The green line shows how the radial strain of the outer layer increases slowly with the internal diameter. This behavior is due to the driving force related to the differences in charge density being reduced with increased internal diameter. The radial expansion is undesirable for actuation as it can be regarded as a loss, being out of phase with the desired actuation direction. To further complement this analysis, we provide additional simulation results in the Supporting Information that isolate the effect of wall thickness on HFDEA performance while keeping the internal diameter fixed.

### 3.3 Optimal Design for HFDEA

Understanding the relationship between fiber geometry and performance is essential for optimizing HFDEAs. Building on the analytical trends explained earlier analysis in Section 3.1 through the a key parameters, 𝛽, which encapsulates the combined effects of internal diameter (𝑖𝑑) and wall thickness (𝑡ℎ) on actuator behavior, we now turn to a numerical evaluation of how these geometric variations influence actuator performance under more realistic operating conditions.

**Figure 10:**
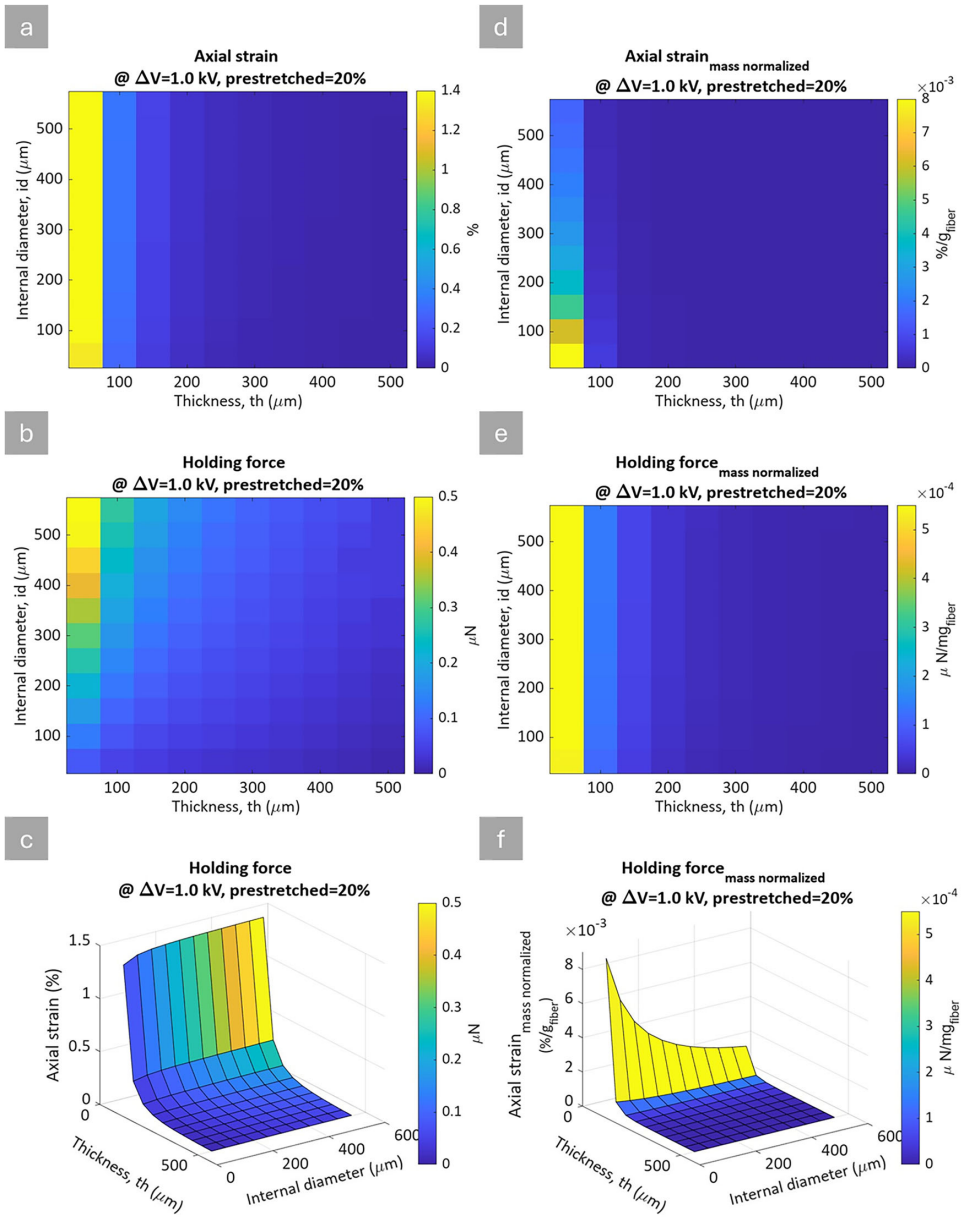
Simulation results under 1 kV applied voltage and 20% pre‐stretched conditions. Notice that subfigures a‐c and d‐f are vertically aligned. a–c) absolute values: (a) heatmap of axial strain as a function of internal diameter (id) and wall thickness (th) (b) heatmap of holding force as a function of id and th (c) 3D plot illustrating the relationship between holding force (color), axial strain (z‐axis), id and th in x and y‐axis; (d–f) mass normalized values: d) heatmap of mass‐normalized axial strain as a function of internal diameter (id) and wall thickness (th) e) heatmap of mass‐normalized holding force as a function of id and th f) 3D plot illustrating the relationship between mass‐normalized holding force (color), mass‐normalized axial strain (z‐axis), id and th in x and y‐axis.

However, it is important to note that this comparison does not investigate the weight of the material required for a given strain, an important factor to be considered. To address this, the subsequent analysis compares absolute (non‐normalized) and mass‐normalized values. The mass normalization analysis was performed by calculating the fiber's mass per 1 mm length. This is calculated by multiplying the elastomer density (0.97 g cm^−3^) by the cross‐sectional area of the fiber and its unit length. **Figure** **10** shows the result of this approach, where the left column (Figure 10a–c) presents absolute values, and the right column (Figure 10d–f) shows the mass normalized values for axial strain and holding force across varying geometries of internal diameter (*id*) and wall thickness (*th*). Further results, including pre‐stretched corresponding values, are provided in Figures S4 and S5 (Supporting Information). Under pre‐stretched conditions, the fiber undergoes geometric changes that influence its mechanical response. As the fiber extends under load, the effective elastic modulus decreases. This reduction in stiffness further contributes to an increase in strain.

While the primary focus of this study is on actuation strain, another important performance indicator, the actuator's holding force (blocking force), is also evaluated. The holding force is the greatest force the actuator can apply when its deformation is restricted, making it essential for efficient actuation design. This factor is challenging to measure for this scale of size, but it is introduced as it is an important component in HFDEA design.

A deeper investigation into the physical mechanisms underlying the blocking force behavior was also conducted. The holding force in HFDEAs originates from the electrostatic attraction between oppositely charged electrode surfaces, balanced by the mechanical stiffness of the elastomer shell. A simplistic expression for the holding force is the Maxwell pressure multiplied by the area over which the pressure is maintained (∼πd_i_L). As the internal diameter increases for a given fiber length, the effective electrostatic surface area grows almost proportionally with the inner diameter. The decrease in thickness leads to a larger electrostatic pressure, thereby increasing the actuator's capacity to generate force under constrained conditions. However, likewise, the mass of the fiber with the same length and thickness scales with the inner diameter, so when comparing the mass‐normalized holding forces, there is no difference between the actuators. In other words, it all comes down to the thickness of the actuator, as for the planar configuration. While these trends are apparent in the 2D heatmaps (Figure 10), we have also included supporting line plots in (Figures S2 and S3, Supporting Information), which isolate the effect of each geometric parameter. These one‐variable simulations help further clarify the sensitivity of the holding force to individual design choices and validate the trends observed in the more comprehensive heatmaps.

The heatmap in Figure 10a illustrates how the axial strain peaks at minimal wall thickness and large internal diameters. This trend aligns with the *β* profile, where thinner walls and larger diameters enhance electrostatic expansion. Similarly, Figure 10b, shows the holding force also peaks at minimal wall thickness and large internal diameters, reinforcing that this geometric configuration benefits actuator performance. The 3D figure in Figure 10c further highlights that both axial strain and holding force maximize with large *id* and thinner *th*. The right column of Figure 10 provides insight into actuator efficiency through mass normalization. When normalized by mass, the axial strain is at its maximum at smaller wall thicknesses and smaller internal diameters, as illustrated in the heatmap of Figure 10d. This suggests that fibers with thinner walls and smaller internal diameters exhibit a larger normalized strain for a given mass, making them more efficient in terms of material usage and when considering the overall actuator design, e.g., fiber bundles. Figure 10e indicates that the mass‐normalized holding force peaks with minimal wall thickness and large internal diameters, similar to the trend observed in absolute holding force. The 3D plot in Figure 10f, illustrates that while larger diameter and thinner walls enhance mass‐normalized holding forces, smaller diameter fibers with thin walls are more efficient for axial strain in terms of material usage.

In conclusion, Figure 10 illustrates the interplay between geometric parameters and the performance of HFDEA actuators under a given applied voltage. Maximizing the absolute axial strain and the holding force involves increasing the internal diameters and reducing the thickness. However, for strain efficiency relative to mass, smaller and thinner fibers are more favorable. This dual approach helps in designing high‐strain actuators while keeping the weight of the actuator low, depending on which requirements are important for a given application.

## 4 Conclusion

For the fiber geometry, it is shown that we generally have at least 2 times the actuation strain achievable for a planar actuator at the same thickness. Furthermore, in the two extremes of small and large inner diameter, we achieve even bigger improvements compared to the planar geometry. These extremes are due to two physical phenomena, namely that at low inner diameter, the difference in charge densities on the two electrodes becomes a main driver for, in particular, movement of the inner surface, contributing strongly to the actuation. Second, large inner diameters give the overall best actuation performance per length unit (not per mass, though) due to the movement being more or less confined to two dimensions only.

While the analytical models provided valuable insights into the strain performance of HFDEAs, they are limited to small‐strain conditions (≤10%). Beyond this range, the strain calculation becomes inaccurate. However, the numerical models addressed this gap by incorporating nonlinear elasticity (Mooney‐Rivlin) and more realistic boundary conditions, offering a better option for exploring HFDEAs' performance in a wider strain range. Despite these limitations, the analytical model remains a powerful tool for identifying general trends and guiding initial design choices.

Experimental validation involved fabricating fibers with different internal diameters and wall thicknesses. The simulations demonstrated that optimal performance is achieved through specific geometric configurations, characterized by greater internal diameters and thinner walls, which enhance both axial strain and holding force. However, smaller and thinner fibers were found to deliver greater strain in terms of material efficiency, emphasizing the importance of balancing overall strain performance and material usage. This trade‐off is governed by the balance between the mechanical stiffness (influenced by geometry and material properties) and the electrostatic forces (dependent on the applied voltage and electrode configuration). The complicated relationships between axial strain, holding force, geometric factors, and applied voltage were visualized using heat maps and 3D graphs.

In summary, HFDEAs outperform conventional planar film designs, particularly when considering higher strain performance, facile integration into complex systems, and efficient material usage are required. This study provides a solid foundation for research and development of HFDEAs, paving the way for their application in advanced soft robotics and other cutting‐edge technologies.

## Conflict of Interest

The authors declare no conflict of interest.

## Author Contributions

S Jafarzadeh—conceptualization, investigation, formal analysis, original draft, review & editing; A L Skov—conceptualization, supervision, investigation, original draft, review & editing, funding acquisition, project administration.

## Supporting information



Supporting Information

## Data Availability

The data that support the findings of this study are available from the corresponding author upon reasonable request.
