# Supplementary material for: Beyond Planar: Enhanced Performance of Hollow Fiber Dielectric Elastomer Actuators
Source: Adv Sci (Weinh). 2025 Jun 27;12(33):e04803. doi: 10.1002/advs.202504803 (PMC12412549; doi:10.1002/advs.202504803)
Supplement: Supplementary file 1 — Supporting Information [file ADVS-12-e04803-s001.docx]

Supporting Information

Beyond planar: enhanced performance of hollow fiber dielectric elastomer actuators

Sina Jafarzadeh, Anne Ladegaard Skov*

This supplementary information provides additional details and data to complement the findings presented in the main article. The supplementary information is organized as follows:

1. General principles of planar actuator modeling

A theoretical overview of planar dielectric elastomer actuators, detailing the basic mechanisms of operation and key equations that describe their behavior.

1. Mechanical characterization

Experimental data on the mechanical properties of the hollow fiber elastomers, including fitting to the Mooney-Rivlin model.

1. Influence of wall thickness at fixed internal diameter on actuation strain

Simulation results showing how increasing wall thickness at fixed internal diameter (100 µm) reduces axial and radial strains due to lower electrostatic pressure and weaker electro-attractive forces.

1. Influence of individual geometric parameters on holding force

Simulation results showing how holding force varies with internal diameter and wall thickness at 1 kV. Larger diameters increase force via greater surface area, while thicker walls reduce force due to lower field strength and higher stiffness.

1. Mass normalized simulation results under 1 kV applied voltage and varying pre-stretched conditions

Simulation results show the relationship between axial strain, internal diameter, and wall thickness under different pre-stretch conditions.

1. Simulation results under 1 kV applied voltage and varying pre-stretched conditions

Further simulation data exploring the impact of pre-stretch on the performance of the actuators, including 3D plots and heatmaps illustrating key performance metrics.

# General principles of planar actuator modeling

Planar dielectric elastomer actuators in the planar figuration function like parallel-plate capacitors, where the separation between the two oppositely charged plates determines the capacitance. The distance between the plates (film thickness, *th*) and the dielectric material influences the capacitance. Due to the flexibility of the dielectric elastomer, the electrostatic forces between the compliant electrodes compress the elastomer in thickness and stretch it in the perpendicular plane, converting electrical energy into mechanical work. This results in expansion in the planar direction as the material’s thickness decreases.

The capacitance (*C*) of the planar film capacitor is defined as the ratio of the charge (*Q*) on each electrode to the potential difference (ΔV) between them:

$C=\frac{Q}{\Delta V}$ S 1

For two plates of area 𝐴 separated by a distance ($z$) by a homogenous material with a dielectric of permittivity $\varepsilon=\varepsilon_{0}\varepsilon_{r}$ , the electric field (𝐸) is uniform and given by:

$E=\frac{\Delta V}{z}$ S 2

Where $\varepsilon_{0}$ is vacuum permittivity constant (8.854×10^−12^ F.m^−1^) and $\varepsilon_{r}$ relative permittivity of the dielectric material. The displacement field (𝐷) in the dielectric material between the plates is:

$D=\varepsilon E=\varepsilon\frac{\Delta V}{z}$ S 3

Since the displacement field (𝐷) represents the charge per unit area (𝜎) on the plates, we have:

$Q=\sigma A=DA=\varepsilon\frac{V}{z}A$ S 4

And by substituting 𝑄 in the capacitance definition, we get:

$C=\frac{\varepsilon A}{z}$ S 5

The stored electrostatic energy ($U$) for a film with opposite charges ($Q$) and ($-Q$) on its surfaces is:

$U=\frac{Q^{2}}{2C}=\frac{{C\Delta V}^{2}}{2}=\frac{{\varepsilon A\Delta V}^{2}}{2z}$ S 6

The stored electrostatic energy represents the work performed on the DEA by the electric field. As a result, it increases the electrostatic force ($F$) exerted on the electrodes. This force can be determined by taking the derivative of the stored electrostatic energy concerning the displacement in the direction of the electrical field along the elastomer’s thickness ($z$). For planar actuators, the electrostatic force can be expressed as:

$F_{z}\text{=}-\frac{\partial U}{\partial z}$ S 7

In response to the applied voltage ($\Delta V$), the electrostatic pressures ($P$) on each electrode can be determined by the electrostatic force acting between the electrodes divided by the respective area of the electrode (A):

$P_{z}\text{=}-\frac{1}{A}\frac{\partial U}{\partial z}$  S 8

from volumetric incompressibility of elastomer, we have:

$d\left( volume \right)=0$ S 9

For planar configuration, the volume is determined by electrode area and the thickness therefor, we get:

$d\left( A*z \right)=0$ S 10

this expression can be expanded as:

$\frac{dA}{A}=-\frac{dz}{z}$ S 11

And finally, solving for $dA$ we get:

$dA=-\frac{dz}{z}A$  S 12

The change in stored electrostatic energy ($dU$) for a change in thickness ($dz$) and in area ($dA$) is derived as:

$dU=\frac{\partial U}{\partial A}dA+\frac{\partial U}{\partial z}dz$  S 13

From **S 6** we can find the following:

$\frac{\partial U}{\partial A}=\frac{{\varepsilon\Delta V}^{2}}{2z}$  S 14

$\frac{\partial U}{\partial z}=-\frac{{\varepsilon A\Delta V}^{2}}{2z^{2}}$  S 15

By inserting **S 14** and **S 15** into **S 13**, we have:

$dU=\frac{{\varepsilon\Delta V}^{2}}{2z}dA-\frac{{\varepsilon A\Delta V}^{2}}{2z^{2}}dz$ S 16

And then by substituting ***S 12***, the expression can be arranged as below:

$dU=-\frac{{\varepsilon\Delta V}^{2}}{2z}\frac{dz}{z}A-\frac{{\varepsilon A\Delta V}^{2}}{2z^{2}}dz=-\frac{{\varepsilon A\Delta V}^{2}}{z^{2}}dz$ S 17

$dU=-\frac{{\varepsilon A\Delta V}^{2}}{z^{2}}dz$ S 18

Then, electrostatic pressure can be calculated from **S 8.** The resulting pressure is directed inward as the electric field compresses the elastomer film. By convention, compressive pressure is considered positive. Therefore, the electrostatic (Maxwell) pressure is expressed as:

$P=\frac{{\varepsilon\Delta V}^{2}}{z^{2}}$ S 19

Given the electric field (𝐸) defined by **S 2**, the electrostatic pressure can be expressed as:

$P={\varepsilon E}^{2}$ S 20

The strain of the elastomer depends on various factors, including boundary conditions, the modulus of elasticity, and the loading. The stain ($s_{z}$) in the thickness direction can be calculated as:

$s_{z}=-\frac{p}{Y}=-\frac{\varepsilon E^{2}}{Y}=-\frac{\varepsilon}{Y}{(\frac{\Delta V}{z})}^{2}$ S 21

${(\frac{\Delta z}{z}\text{)}}_{film}\text{=}-\frac{\varepsilon}{Y}{(\frac{\Delta V}{z})}^{2}$ S 22

In the planar film, the area of the electrode can be expressed as:

$A=xy , x=y \to A=x^{2}$ S 23

By taking the differential, we have:

$dA=2xdx$ S 24

Which can be arranged as follows:

$\frac{dA}{A}=\frac{2xdx}{A}\to\frac{dA}{A}=\frac{2dx}{x}$ S 25

By comparing **S 11** and **S 24**, we can have:

$\frac{2dx}{x}=-\frac{dz}{z}$ S 26

Thus, in-plane strain can be derived as:

${(\frac{\Delta x}{x})}_{film}=\frac{1}{2}{(\frac{\Delta z}{z}\text{)}}_{film}$ S 27

${(\frac{\Delta x}{x})}_{film}={(\frac{\Delta y}{y})}_{film}=\frac{\varepsilon}{2Y}\frac{{\Delta V}^{2}}{z^{2}}$ S 28

Considering the small strain (≤10%) condition, the actual dimension of the film (z) in **Equations S 22** and **S 28** can be approximated by the initial thickness (*th*) to simplify the equations and avoid complications arising from higher-order nonlinearities. Thus, the out-of-plane and in-plane strains can be expressed as follows:

${(\frac{\Delta z}{z}\text{)}}_{film}\text{=}-\frac{\varepsilon}{Y}\left( \frac{\Delta V}{th} \right)^{2}$ S 29

${(\frac{\Delta z}{z}\text{)}}_{film}\text{=}\frac{\varepsilon}{2Y}{(\frac{\Delta V}{th})}^{2}$ S 30

# Mechanical characterization of the elastomer

The mechanical properties of the hollow fiber elastomers were characterized using the Mooney-Rivlin model. The strain energy density function *W* for the Mooney-Rivlin model is expressed as:

$W=C_{10}(I_{1}-3)+C_{01}(I_{2}-3)$ S 29

where $C_{10}$ and $C_{01}$ are the material constants or parameters of the model, which were determined from experimental data to ensure accurate simulation outcome (**Figure S2,**). The constants$I_{1}$ and $I_{2}$ represent the first and second invariants of the Cauchy-Green deformation tensor, crucial for understanding the elastomer's behavior under strain. The equations following define these invariants:

$I_{1}={\lambda_{1}}^{2}+{\lambda_{2}}^{2}+{\lambda_{3}}^{2}$ S 30

$I_{1}=({\lambda_{1}}^{2}{\lambda_{2}}^{2})+({\lambda_{2}}^{2}{\lambda_{3}}^{2})+({\lambda_{1}}^{2}{\lambda_{3}}^{2})$ S 31

Here, $\lambda_{1}$, $\lambda_{2}$ and $\lambda_{3}$ are the principal stretch ratios, which describe the deformations along the principal axes of the material. These ratios explain how the material stretches or compresses in response to applied forces. The calculated values of $C_{10}$ and $C_{01}$ are 64.5 kPa and 17.3 kPa, respectively.


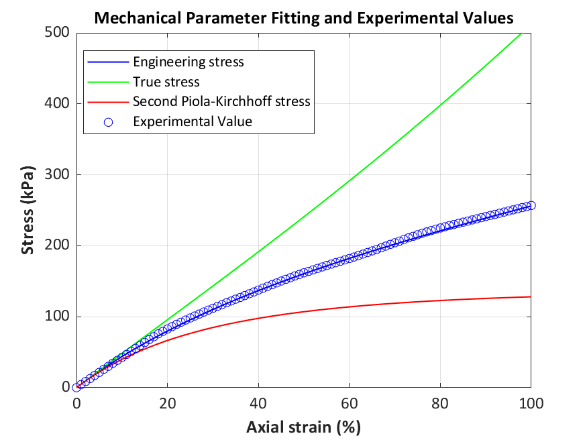


**Figure S1**. Experimental data (blue circles) and numerical fitting: Mechanical characterization of the hollow fiber elastomer, including the Mooney-Rivlin fitting. The plot illustrates the stress-strain response with four distinct curves: engineering stress, true stress, second Piola-Kirchhoff stress, and experimental data. The engineering stress curve represents the 2D Mooney-Rivlin fitted model, providing a clear comparison between theoretical predictions and experimental observations.

The pre-stretch in the modeling is implemented by introducing a load corresponding to the desired pre-stretch strain at one end of the fiber while the other end remains fixed. Once pre-stretching is established, the actuation strain is determined by comparing the deformed state under actuation with the initial, non-actuated pre-stretched configuration.

# Influence of wall thickness at fixed internal diameter on actuation strain

**Figure S2** presents additional simulation results evaluating the influence of wall thickness on the actuation strain behavior of HFDEAs, while maintaining a constant internal diameter of 100 µm. The figure provides comparative strain profiles, including axial strain, internal boundary radial strain, and external boundary radial strain, at various wall thicknesses. The results show a consistent decrease in all strain components with increasing wall thickness. This reduction is primarily due to the decreased electrostatic pressure and the weaker electro-attractive interaction between inner and outer surfaces as the distance between them increases. These insights help decouple the influence of wall thickness from that of internal diameter, offering more practical design guidelines.


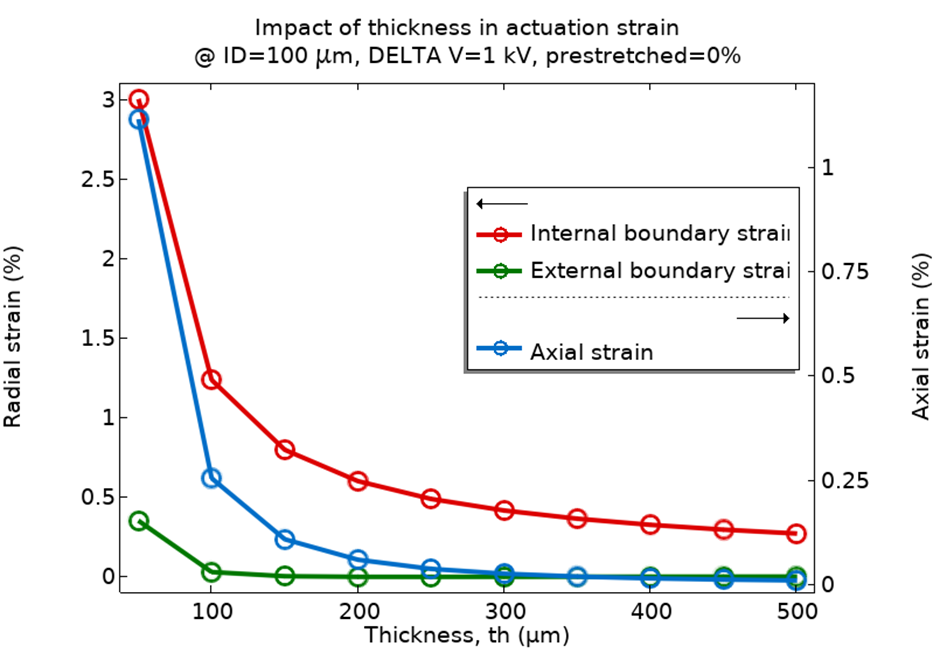


**Figure S2**. Simulation results of HFDEAs at 1 kV applied voltage showing the effect of wall thickness on strain behavior at a fixed internal diameter of 100 µm. Displayed are the axial strain (blue), internal radial strain (red), and external radial strain (green).

# Influence of individual geometric parameters on holding force

**Figure S3** presents one-dimensional simulation results illustrating the effect of individual geometric parameters, internal diameter and wall thickness, on the holding force of HFDEAs under a fixed applied voltage of 1 kV and 0% pre-stretch.

In Figure S3a, the internal diameter is varied while maintaining a constant wall thickness of 100 µm. The holding force shows a monotonic increase, confirming the role of increased electrostatic surface area in enhancing actuation force.

In Figure S3b, the wall thickness is varied with a fixed internal diameter of 100 µm. As expected, the holding force drops significantly with increasing thickness due to both reduced electric field strength and increased mechanical stiffness.

These results complement the multidimensional maps presented in the main text and offer a more focused analysis that may assist in fine-tuning actuator geometries for targeted applications.


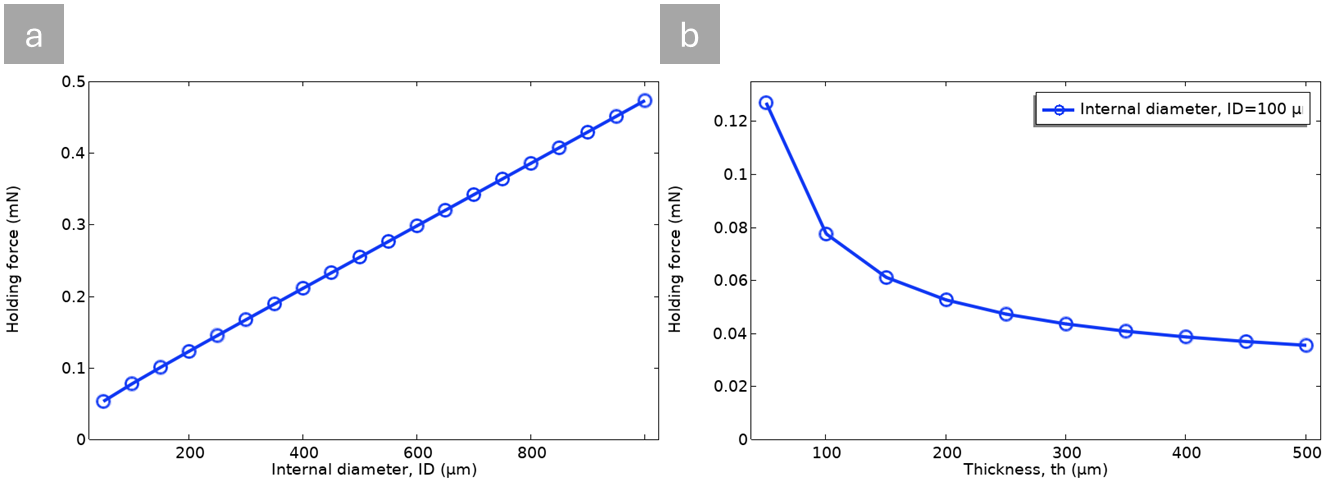


**Figure S3**. Simulation results of holding force in HFDEAs at 1 kV and 0% pre-stretch. (a) holding force versus internal diameter (ID) for a constant wall thickness (100 µm). (b) holding force versus wall thickness (th) at a fixed internal diameter of 100 µm.

# Simulation results under 1 kV applied voltage and varying pre-stretched conditions

**Figure S4** shows additional simulation results under a 1 kV applied voltage, focusing on the effects of pre-stretch. These include heat maps and 3D plots similar to those in the previous section, highlighting how pre-stretch influences the performance of the hollow fiber dielectric elastomer actuators.


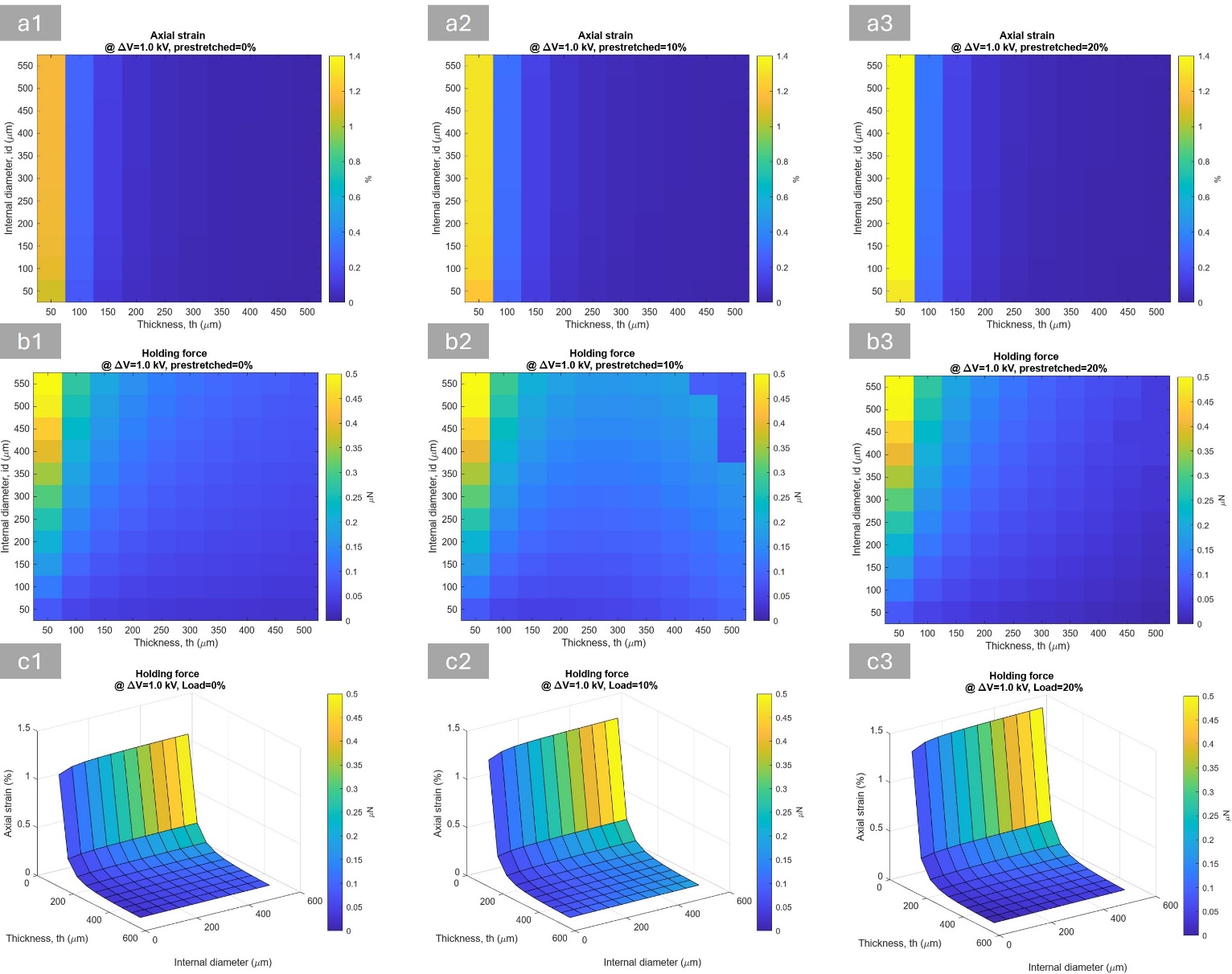


**Figure S4**. Simulation results under 1 kV applied voltage and varying pre-stretched conditions (left column to right 0,10% and 20% pre-stretched) (a1-3) heatmap of axial strain as a function of internal diameter (id) and wall thickness (th) (b1-3) heatmap of holding force as a function of id and th (c1-3) 3D plot illustrating the relationship between holding force (color), axial strain (z-axis), id and th in x and y-axis.

# Mass normalized simulation results under 1 kV applied voltage and varying pre-stretched conditions

Simulation results are presented under a 1 kV applied voltage with varying pre-stretch conditions (0%, 10%, and 20% pre-stretch). These results are mass-normalized and depicted as heat maps of axial strain and holding force, as well as 3D plots showing the relationship between holding force, axial strain, internal diameter, and wall thickness. **Figure S5** provides a detailed visualization of these simulation results.


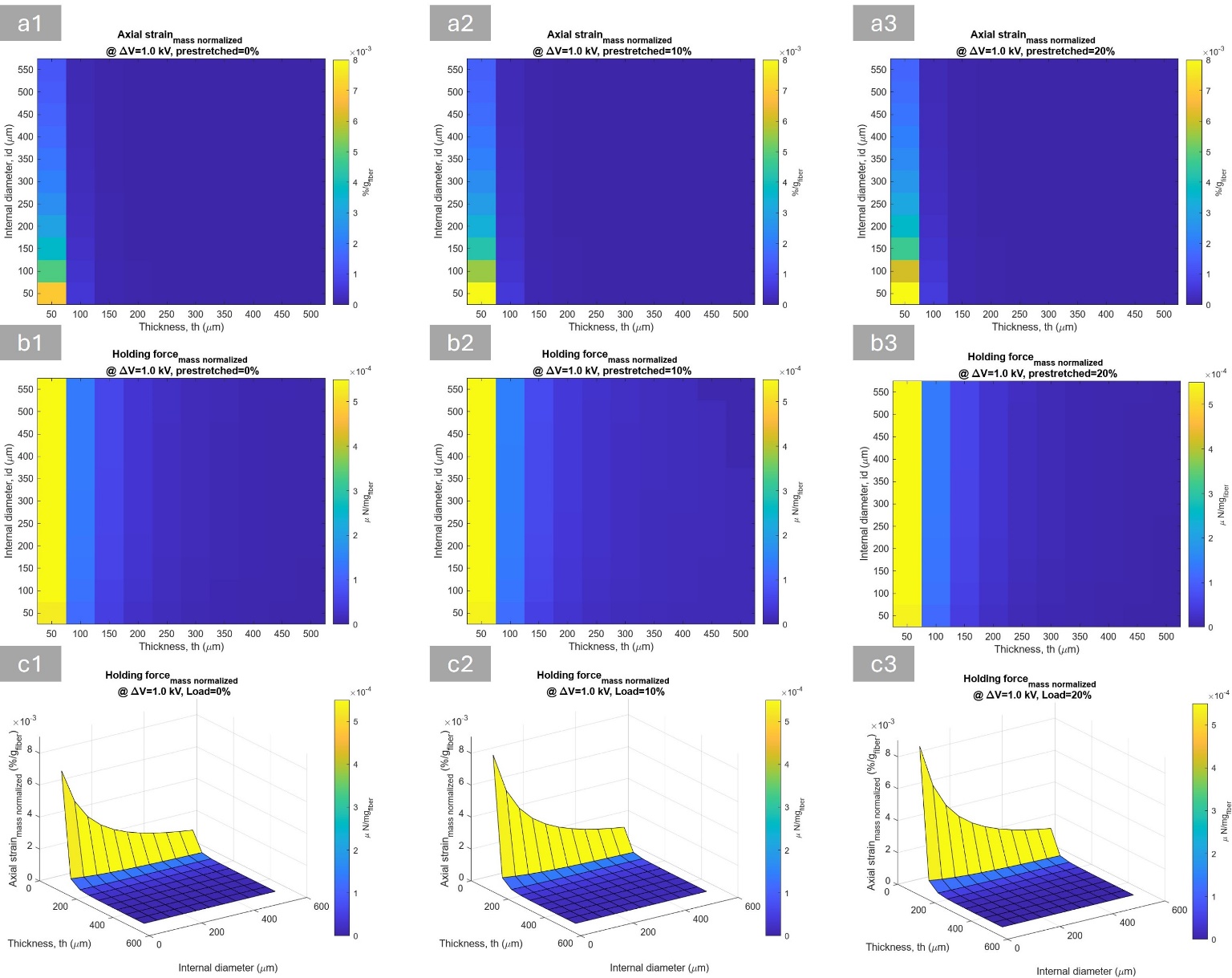


**Figure S5**. Simulation results: Mass normalized results under 1 kV applied voltage and varying pre-stretched conditions (left column to right 0,10% and 20% pre-stretched) (a1-3) heat map of axial strain as a function of internal diameter (id) and wall thickness (th) (b1-3) heatmap of holding force as a function of id and th (c1-3) 3D plot illustrating the relationship between holding force (color), axial strain (z-axis), id and th in x and y-axis.
